# Contamination Status and Health Risk Assessment of 73 Mycotoxins in Four Edible and Medicinal Plants Using an Optimized QuEChERS Pretreatment Coupled with LC-MS/MS

**DOI:** 10.3390/toxins17020052

**Published:** 2025-01-23

**Authors:** Xiaojing Huang, Rui Feng, Qing Hu, Xiuhong Mao, Heng Zhou

**Affiliations:** Shanghai Institute for Food and Drug Control, Shanghai 201203, China

**Keywords:** mycotoxins, edible and medicinal plants, QuEChERS, UHPLC-MS/MS, contamination status, health risk assessment

## Abstract

The current status of multi-mycotoxin contamination in edible and medicinal plants demands urgent development of high-throughput analytical methods for mycotoxin detection. In this study, a reliable and sensitive method for the simultaneous analysis of 73 mycotoxins was established and successfully applied to detect mycotoxins in 260 samples of four dual-purpose plants (lotus seed, coix seed, licorice root, and dried tangerine peel). Sample preparation involved optimized QuEChERS (Quick, Easy, Cheap, Effective, Rugged, and Safe) extraction combined with liquid–liquid extraction purification, and an enhanced ion pair library was established to reduce matrix interference and improve the method’s universality. Method validation demonstrated recovery rates ranging from 61.6% to 118.6% for all compounds, with relative standard deviations (RSDs) below 15%. The limits of detection (LODs) and quantification (LOQs) ranged from 0.25–12.25 μg/kg and 0.5–25 μg/kg, respectively. Based on the contamination analysis and health risk assessment using Margin of Exposure (MOE) and Hazard Index (HI) methods, we found that multi-mycotoxin contamination is highly prevalent in edible and medicinal plants, with different components being susceptible to invasion by distinct fungal genera. Seed-type plants showed high susceptibility to Aspergillus (53.3%) and Fusarium (22.2%) contamination, with MOE values below 10,000 for aflatoxins indicating potential health risks. Physical state and good storage conditions significantly influenced contamination levels, with fragmented samples showing substantially higher mycotoxin levels. Additionally, mycotoxins with associated biosynthetic metabolic pathways were frequently detected simultaneously in highly contaminated samples. Based on these findings, we recommend implementing strict moisture control during storage, maintaining intact product form where possible, and establishing comprehensive supplier qualification systems. This study provides valuable reference for monitoring mycotoxin contamination in similar plants.

## 1. Introduction

Mycotoxins, as secondary metabolites produced by fungi, readily contaminate various food matrices, including foodstuffs, oils, and traditional Chinese medicines (TCMs) [1,2,3]. To date, over 400 mycotoxins have been identified and isolated, with several, such as aflatoxins and ochratoxins, being demonstrated to possess severe toxic effects, including the induction of hepatocellular carcinoma and various diseases affecting the urinary and gastrointestinal systems [1,4,5]. Furthermore, co-contamination by multiple mycotoxins may result in synergistic toxic effects. For instance, the combined toxicity of aflatoxin B_1_, zearalenone, and deoxynivalenol mixture exhibits enhanced hepatotoxicity in rat hepatocytes compared to their individual effects. Furthermore, certain matrices are susceptible to contamination by both masked and emerging mycotoxins [6,7,8]. These circumstances pose significant threats to public health [9,10,11]. Consequently, numerous countries and organizations have established limits for mycotoxins. For example, the European Commission recently issued Commission Regulation (EU) No 2023/915 of 25 April 2023 on the maximum levels for certain contaminants in food, repealing Regulation (EC) No 1881/2006, which now stipulates maximum levels for sixteen mycotoxins (including three newly added ones) in foodstuffs.

Globally, over two billion people rely on TCMs for their health benefits and, with the increasing emphasis on health and wellness, the consumption of edible TCMs has risen substantially [12,13,14,15]. However, regulatory standards for mycotoxin limits in plants lag behind those for food products, primarily due to insufficient detection methods, contamination data, and related risk assessments. Current high-throughput mycotoxin detection methods predominantly focus on food matrices, typically employing a QuEChERS or “dilute and shoot” method for sample preparation. For instance, Michael Sulyok et al. developed a method combining direct extraction with LC-MS/MS to determine 39 mycotoxins in wheat and corn [16,17]. Similarly, Ádám Tölgyesi et al. developed a novel LC-MS/MS multi-method for the simultaneous determination of 295 food contaminants in cereals, including 266 pesticides, 12 mycotoxins, 14 alkaloid toxins, and three Alternaria toxins [18,19,20].

Compared to food matrices, edible and medicinal plants present unique analytical challenges as dried materials containing numerous metabolites, some structurally similar to mycotoxins. This complexity creates significant matrix interference for trace mycotoxin detection, affecting methods’ sensitivity and accuracy. When applying food-based high-throughput detection methods to plants, several limitations emerge. For example, Zhao et al. observed significant matrix interference affecting mycotoxin recovery rates in nutmeg, galangal, and coix seeds using a “dilute and shoot” method [21]. When applying the QuEChERS methodology, certain aminopropyl (NH_2_), primary secondary amine (PSA) cleanup sorbents, while effective at removing fatty acids and organic acids, can inadvertently adsorb acid-sensitive toxins containing carboxyl groups, resulting in reduced recovery rates [21,22]. To minimize matrix interference and achieve higher recovery rates and sensitivity, combining different sample preparation methods to leverage their respective advantages presents a viable solution [23]. For instance, Nouri and Sereshti developed a rapid method combining SPE with DLLME for determining aflatoxins in soybeans [24].

Currently, research on mycotoxin contamination distribution patterns primarily focuses on food matrices and typically examines only a few specific mycotoxins or mycotoxin classes. For example, Abirami Ramu Ganesan et al. investigated the distribution patterns of Ochratoxin A and deoxynivalenol in agricultural products and related foods, while Sun et al. studied the contamination profiles of aflatoxins, ochratoxins, and fumonisins in Chinese rice [25,26]. However, there is limited research exploring the potential correlations between edible and medicinal plants and their specific mycotoxin contamination.

Chemical compounds with interactive effects may exhibit lower or higher toxic effects compared to individual substances, necessitating cumulative exposure risk assessment for multiple chemical compounds [27]. Consequently, cumulative exposure assessment methods are more appropriate for evaluating multi-mycotoxin contamination in matrices. The main cumulative exposure assessment methods include the Margin of Exposure (MOE), Hazard Index (HI), Relative Potency Factor (RPF), and Point of Departure (POD). The RPF requires similar toxicological targets, exposure routes, and duration among components in chemical mixtures, making it unsuitable for assessing contamination by diverse mycotoxin types. Additionally, there is no internationally standardized evaluation method for the POD approach. Therefore, the MOE and HI are currently the primary methods employed for cumulative mycotoxin exposure assessment. The European Food Safety Authority (EFSA) has specifically identified the MOE as the most suitable approach for evaluating genotoxic carcinogens [28]. For example, Zhang et al. applied both MOE and HI methods to assess different mycotoxins in dual-purpose plants such as coix seed and lotus seed based on their toxicity profiles [29]. Similarly, Lu et al. utilized the HI method to evaluate 31 mycotoxins in six edible and medicinal plants [30].

This study encompasses 73 mycotoxins produced by major toxigenic fungi, including *Fusarium*, *Claviceps*, *Alternaria*, and *Penicillium* species [2] (Table 1). The coverage extends to regulated mycotoxins, their associated masked forms, and emerging mycotoxins such as Enniatins and Beauvericin, aiming to provide comprehensive contamination data. Considering exposure levels, four commonly used edible and medicinal plants were selected as research subjects: lotus seed (LS), coix seed (CS) [14,31,32], licorice root (LR), commonly used as a sweetener [33], and dried tangerine peel, named “chenpi” in China (CP), often preserved as candied fruit [34]. CP can be stored for decades and its source material (tangerines) is particularly susceptible to fungal contamination.

This study developed a robust, high-throughput analytical method for these 73 mycotoxins by combining optimized QuEChERS with liquid–liquid extraction and establishing a more comprehensive ion pair library. This method was successfully applied to four edible and medicinal plants, enabling a detailed analysis of their contamination levels and characteristics. A risk assessment for Chinese populations was conducted using both the MOE and HI approaches. The findings provide valuable reference data for mycotoxin risk assessment in edible and medicinal plants and the development of relevant regulatory standards.

## 2. Results and Discussion

### 2.1. Method Optimization

#### 2.1.1. Optimization of UHPLC-MS/MS Conditions

**Figure 1:**
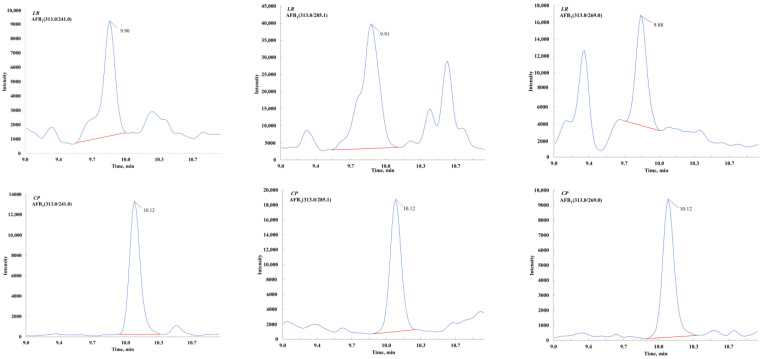
The optimal ion pairs in different plants for certain mycotoxin.

At the beginning of this study, mass spectrometric conditions from previous literature were referenced, including the detection of 191 mycotoxins reported by Elisabeth Varga et al. and 41 mycotoxins reported by Ann-Kristin Rausch et al. [35,36]. When applied to herbal medicine matrices, significant matrix interference was observed near some target peaks. However, this issue could be effectively resolved by modifying the MRM transitions. This demonstrates that differences in matrices require the consideration of ion pair specificity rather than merely ion response intensity. Subsequently, standard solutions of 73 mycotoxins (dissolved in 50% methanol at 500 ng/mL) were individually injected into the MS/MS system at a constant flow rate of 5 μL/min. The Analyst 1.5.1 software was used to compare and select the optimal precursor and product ions. For each mycotoxin, 3–5 ion pairs were optimized to enhance method applicability (Table 2). As shown in Figure 1, for AFB_1_ quantification in licorice ([M+H]^+^), the optimal MRM transitions were 313.0 > 241.0 and 313.0 > 269.0, while for AFB_1_ in tangerine peel, they were 313.0 > 241.0 and 313.0 > 285.1. Notably, although the product ion transition 313.0 > 285.1 exhibited higher intensity, undesirable interference peaks were observed near the AFB_1_ peak (*m*/*z* 313.0 > 285.1) in the LR. The establishment of a more comprehensive MRM transition library significantly improved the method’s versatility. To our knowledge, such an extensive ion pair spectral library for more than 70 mycotoxins has not been previously reported.

Due to the significant matrix effects in Chinese herbal medicines and the large number of target analytes, the optimized chromatographic conditions were designed to achieve maximum response intensity and optimal resolution for all analytes. Following Elisabeth Varga’s approach, chromatographic separation was performed in both positive and negative ionization modes [36]. Since more mycotoxins were separated in the positive mode, methanol (MeOH) was selected as the organic phase due to its relatively weaker elution strength, enabling better separation. Various modifiers, including formic acid, acetic acid, ammonium formate, and ammonium acetate, were evaluated to enhance ionization efficiency. The addition of 0.4% formic acid improved the response of many mycotoxins, particularly fumonisins and ochratoxins. Ammonium formate supported better peak shapes through the formation of [M+NH_4_]^+^ adducts. The optimal concentration was determined to be 2 mM, as higher concentrations (5 mM) led to ionization suppression (e.g., for ochratoxin A). In negative mode, with only 9 mycotoxins being detected, switching the organic phase from methanol to acetonitrile improved peak shapes and enhanced sensitivity without requiring modifiers. Additionally, the liquid chromatographic gradient, column temperature, and flow rate were optimized. The final mobile phases consisted of water–acetonitrile (A/B) with 0.4% formic acid and 2 mM ammonium formate for the positive mode and water–acetonitrile (A/B) for the negative mode. Based on previous research, a core-shell column (Poroshell EC-C18) was selected for its low column pressure and superior separation performance [37]. Although separating the positive and negative ionization modes sacrificed some analytical efficiency, this approach provided a better resolution when analyzing edible and medicinal plant samples, avoiding interference from matrix components and achieving a higher sensitivity. This method demonstrates broader applicability across similar matrix types.

#### 2.1.2. Optimization of Sample Preparation

In 2006, Sulyok et al. first developed an LC-MS/MS method for multi-mycotoxin determination, using direct dilution to analyze 39 mycotoxins in cereals [17]. However, the applicability of this simplified method to plants remained uncertain. We selected LR as the model sample for preparation optimization due to its significant matrix interference. Accuracy evaluation of the “dilute and shoot” method was performed using spiked LR samples (mixed standard solution added to blank LR samples, left overnight at room temperature in a fume hood to better simulate actual mycotoxin contamination). Results indicated that the extraction solvent (acetonitrile/water/acetic acid, 79:20:1, *v*/*v*/*v*) was not compatible with all mycotoxins and matrix interference affected accurate quantification. Alternative extraction solvents were explored to enhance the selectivity and reduce interference, comparing extraction systems composed of formic acid, acetic acid, or citric acid buffer–acetonitrile. As no significant differences were observed among these systems, we maintained the “dilute and shoot” method’s extract solvent system for operational simplicity.

**Figure 2:**
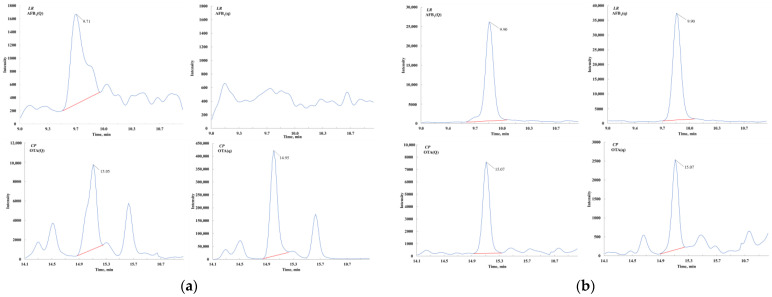
Comparation of chromatograms for the salt-out procedure. (**a**) Chromatography of quantitative (Q) and qualitative (q) ion pairs for sample without salt-out procedure; (**b**) chromatography of quantitative (Q) and qualitative (q) ion pairs for sample with salt-out procedure.

**Figure 3:**
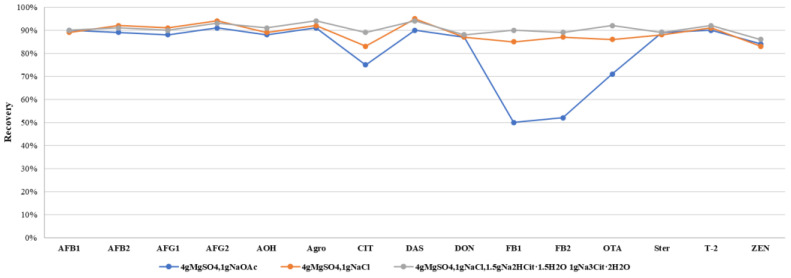
Recoveries of representative mycotoxins in different salt package.

The salting-out step in QuEChERS is commonly used to remove some polar impurities, organic compounds, and proteins. To address the complexity of edible and medicinal plants, we introduced a simplified salting-out step to reduce matrix interference which proved effective in three tested plant matrices (Figure 2). Further comparison of sodium chloride, sodium acetate, and sodium citrate salt packets revealed that anhydrous sodium citrate stabilized solution pH, improving recovery rates of acid-sensitive mycotoxins by 5–8%, consistent with expectations (Figure 3). Conversely, sodium acetate decreased acidity, causing some losses of these mycotoxins.

Innovatively, unlike conventional QuEChERS, we separated the extract from the matrix before adding the aqueous solution for salting-out to minimize the co-extraction of interferents. The effects of water and 5% formic acid solution on mycotoxin recovery were investigated, with a 5% formic acid solution yielding satisfactory recovery rates (70–120%) for most of the mycotoxins.

A challenging issue arose with LS samples, which formed white precipitates during 4 °C storage after processing, affecting measurement accuracy and necessitating effective cleanup. Given the high content of starch, protein, and lipids in lotus seeds, various dispersive solid-phase extraction (d-SPE) sorbents were evaluated, including graphitized carbon black (GCB), enhanced matrix removal-lipid (EMR-Lipid), octadecyl silane (C18), aminopropyl (NH_2_), primary secondary amine (PSA), silica (Si), neutral aluminum oxide (Al-N), carboxymethyl (CBA), diethylaminopropyl (DEA), and cyanopropyl (CN-U). These sorbents were combined with MgSO_4_ (100 mg:900 mg), but none met the requirements due to their poor recovery of important mycotoxins or failure to resolve precipitation issues.

Inspired by Hyun-Deok Cho et al.’s work using hexane for preliminary lipid removal before immunoaffinity column cleanup [22], we modified the approach using cyclohexane. Adding 12.0 mL cyclohexane to 6.0 mL extract significantly improved the precipitation issue while minimally affecting mycotoxin recovery, with only 1–2 mycotoxins showing losses around 7.8% and others below 1.8%. Ultimately, liquid–liquid extraction with cyclohexane was adopted as the cleanup method.

### 2.2. Method Validation

Method validation was performed on three different edible and medicinal plants (LS, LR, and CP), evaluating key analytical parameters including the linearity, accuracy, limits of detection (LOD), limits of quantification (LOQ), and precision. The comprehensive validation data are summarized in Table A1, Table A2 and Table A3.

#### 2.2.1. Linearity

Due to matrix effects exceeding ±20% for most mycotoxins, matrix-matched calibration curves were necessary for accurate quantification. Blank sample extracts after nitrogen evaporation were reconstituted with 0.5 mL acetonitrile, followed by the addition of varying amounts of mixed standard stock solutions. The solutions were then made up to 2 mL with solvent (acetonitrile/water/acetic acid, 20:79:1). Three concentration ranges were prepared: G1 (0.1, 0.5, 1, 5, 10, 20, and 50 ng/mL), G2 (0.5, 2.5, 5, 25, 50, 100, and 250 ng/mL), and G3 (2.5, 12.5, 25, 125, 250, 500, and 1250 ng/mL). Calibration curves were constructed using peak area versus concentration relationships. All mycotoxins demonstrated good linearity with correlation coefficients (r) greater than 0.998.

#### 2.2.2. Method Limit of Quantification (LOQ) and Limit of Detection (LOD)

Spiking experiments were conducted to determine the method’s quantification limits (LOQs) for each matrix. At spiking levels of 0.5 μg/kg (calculated as AFB1), LS and CP samples met requirements for signal-to-noise ratio, recovery, and precision. However, LR samples required a higher LOQ of 1.0 μg/kg (calculated as AFB1), which better reflected actual sample conditions. The LOQs for the three matrices ranged from 0.5 to 25.0 µg/kg, as shown in Table A1, Table A2 and Table A3. Despite using generic extraction and cleanup procedures, the method achieved lower LOQs for several mycotoxins compared to existing reports [29,38]. The LOQs were significantly below the maximum residue limits (MRLs) set by Commission Regulation (EU) No 2023/915. For example, the LOQ for FB1 and FB2 was 2.5 µg/kg, well below the MRL of 200 µg/kg, demonstrating the method’s suitability for regulatory monitoring of these edible and medicinal plants. Limits of detection (LODs) were determined at spiking levels of 0.25 μg/kg (calculated as AFB1) for LS and 0.5 μg/kg (calculated as AFB1) for CP and LR.

During the method’s development, matrix interference for certain mycotoxins in several plants remained unresolved. Consequently, some mycotoxins, such as tenuazonic acid, were excluded from the final method and require further optimization.

#### 2.2.3. Method Accuracy and Precision

In the absence of certified reference materials, the method’s accuracy was evaluated using recovery rates (obtained by spiking known amounts of analytes into blank matrices). Recovery studies were performed at three concentration levels in three blank matrices (*n* = 6): 1.0 μg/kg (Level 1), 5.0 μg/kg (Level 2), and 10.0 μg/kg (Level 3) (calculated as AFB1). The recovery rates for the 73 target analytes ranged from 61.6% to 116.4%, with RSDs less than 14.9%. These results largely comply with European Commission Regulation (EC) No 401/2006, indicating the satisfactory accuracy and precision of the method.

### 2.3. Mycotoxin Contamination of Edible and Medicinal Plants

The established analytical method was applied to analyze 260 batches of four different edible and medicinal plants to characterize their mycotoxin contamination patterns and summarize the distinct contamination characteristics across different plants.

#### 2.3.1. Lotus Seed (LS)

Lotus seeds have a 7000-year history as a vegetable, functional food, and medicinal herb. China is the world’s largest lotus root cultivator and consumer, with a cultivation area of 200,000 hectares [31]. By 2017, Fujian Province’s annual lotus seed production reached 12,205 tons, contributing approximately 1.8 billion RMB to the country’s GDP [39].

**Figure 4:**
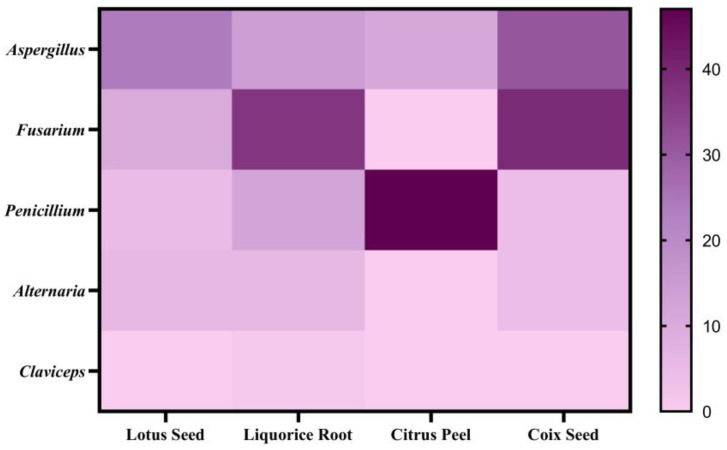
Distribution of susceptible fungi types of four plants.

**Figure 5:**
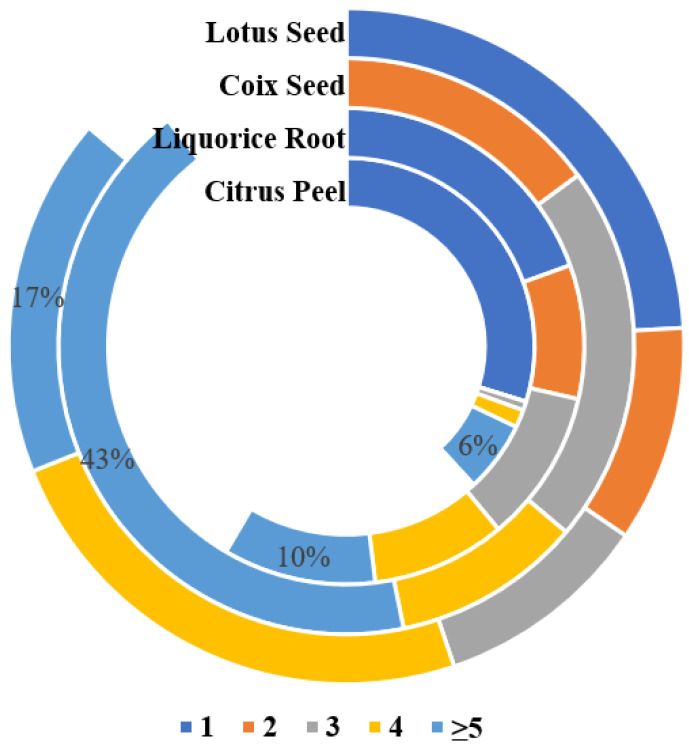
Content percentage of co-occurrence number of mycotoxins in four matrices.

**Figure 6:**
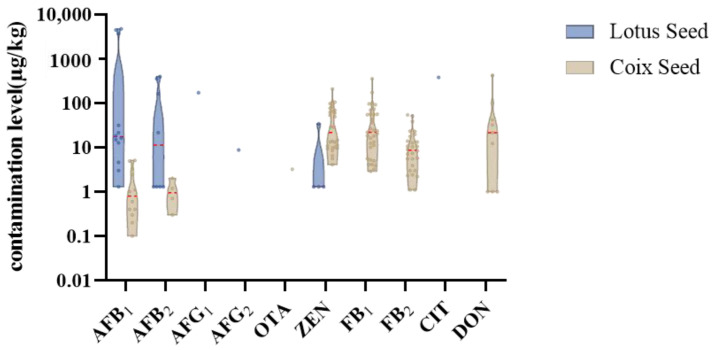
Comparison of the concentration of the regulated mycotoxins in lotus seed and coix seed.

This study analyzed twenty-nine LS samples, including nine special samples (LS28-36): fresh powder (LS31), moldy powder (LS35), discolored powder (LS36), three farm-cultivated powders (LS32-34), and three commercial medicinal samples (LS28-30). In total, 17 mycotoxins were detected in the samples (Table A4), with an 86.2% detection rate, primarily produced by *Aspergillus* species (Figure 4 and Figure 5). Notably, LS showed the highest aflatoxin contamination rate (41.4%) among the four studied plants, at 34.5%, exceeding the Chinese Pharmacopoeia (Ch.P) limits. Three samples—the moldy, discolored, and one farm-cultivated sample—contained AFB_1_ levels up to 4000 μg/kg (Figure 6), indicating rapid aflatoxin accumulation in deteriorated lotus seeds to alarming levels.

The data revealed that highly aflatoxin-contaminated samples frequently contained related metabolites such as AFM_1_, AFM_2_, Ster, and O-m-ster. Interestingly, AFM_1_ and AFM_2_, previously reported only in milk as AFB_1_ metabolites in animals, had never been detected in herbs and spices [40], suggesting possible non-animal AFB_1_ metabolism pathways worthy of further investigation. Additionally, CPA levels exceeded 10,000 μg/kg in these samples, confirming previous reports of AF-CPA co-occurrence and increased toxicity risks [41,42]. Research suggests CPA may serve as a fungal colonization signal molecule, related to its calcium ion-inhibitory activity [43]. Our finding of CPA as the sole mycotoxin in fresh lotus seeds partially supports this hypothesis.

**Figure 7:**
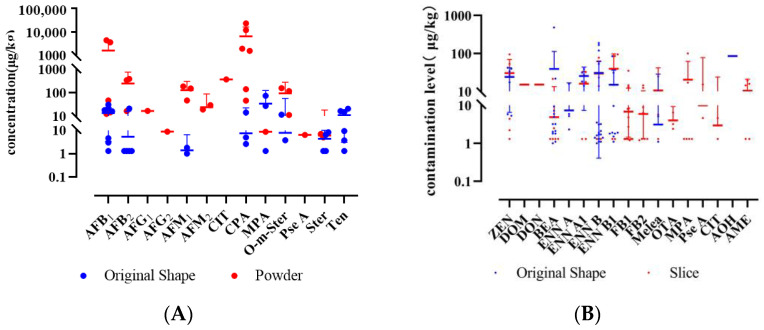
Comparison of mycotoxin levels (mean with 95% CI) between intact and processed forms of LS (**A**) and LR (**B**).

**Figure 8:**
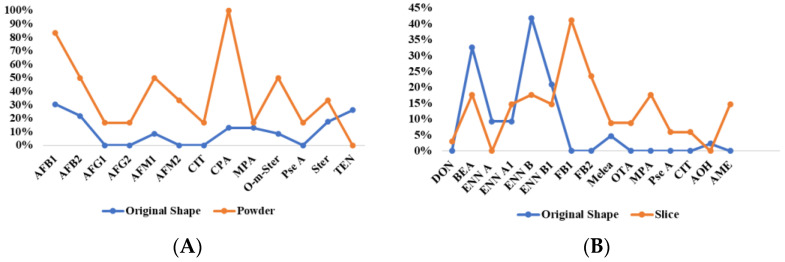
Comparison of mycotoxin detection rates between intact and processed forms of LS (**A**) and LR (**B**).

The significance analysis of mycotoxin contamination in LS of different forms was conducted using the Mann–Whitney U test. Statistical analysis revealed significantly higher detection rates of AFB_1_, AFB_2_, AFM_1_, AFM_2_, CIT, CPA, and O-m-Ster in powder form compared to the original form (*p* < 0.05). No significant differences were observed in the contamination levels of other mycotoxins between the two forms (Figure 7 and Figure 8). The average increase in detection rates was calculated to be 24.7%. Additionally, only discolored lotus seeds contained CIT, a nephrotoxic mycotoxin produced by *Aspergillus*, *Penicillium*, or related fungi, possibly explaining the color change. Therefore, appearance may serve as an important quality indicator for lotus seeds.

#### 2.3.2. Licorice Root (LR)

LR, derived from the dried roots and rhizomes of *Glycyrrhiza uralensis* Fisch, *G. glabra L.*, or *G. inflata* Bat., appears in approximately 60% of TCM prescriptions due to its complementary properties [37]. As one of China’s most widely used herbs, LR is included in multiple pharmacopeias, including Chinese, Korean, European, and United States Pharmacopeias, due to its widespread global use for its sweet taste.

Among 77 samples, 18 mycotoxins were detected, with an overall detection rate of 59.7% (Table A5). The concerning OTA showed a low detection rate of 3.9%, with no samples exceeding the European Pharmacopoeia 11.0 limit (20 μg/kg), while ZEN was detected in 15.6% of samples, indicating potential risks. Contrary to previous reports of high OTA occurrence in licorice [44], 47 samples (LR31-77) from five Chinese regions (Xinjiang, Inner Mongolia, Gansu, Jilin, and Ningxia) showed no OTA contamination, suggesting a possible geographical difference between European and Chinese cultivation regions. Using the same Mann–Whitney U test, sliced LR showed higher contamination levels and detection rates of regulated mycotoxins (FB_1_, FB_2_, OTA, MPA, Pse A, CIT, and AME) compared to raw materials (*p* < 0.05), with increases of 3.7% to 42.1% (Figure 7 and Figure 8).

ENNs and BEA were the predominant contaminants in LR, typically co-occurring due to their similar chemical structures produced by *Fusarium* species. The four enniatins consistently showed a concentration pattern of ENN B > ENN B1 > ENN A1 > ENN A. Research suggests that different *Fusarium* species preferentially incorporate specific amino acids to biosynthesize certain ENNs, explaining why some ENNs can only be isolated from specific fungal strains [45]. The consistent concentration pattern observed in this study suggests contamination by a single *Fusarium* species.

This study analyzed 47 batches of GR samples (GR31-77) that were collected and processed between 2015 and 2020. All samples were maintained in a temperature-controlled storage facility (≤20 °C). Statistical analysis using the Kruskal–Wallis test (*α* = 0.05) revealed no significant temporal differences in mycotoxin levels, with the exception of beauvericin (BEA), enniatin A (ENN A), and enniatin A1 (ENN A1). This indicates that strict collection and storage management with controlled environmental conditions effectively reduces mycotoxin contamination in LR.

#### 2.3.3. Dried Tangerine Peel (CP)

CP, derived from mature fruit peels of *Citrus reticulata* Blanco and its cultivars, is not only one of the most renowned TCMs but also serves as an ingredient in fermented foods [34]. Studies suggest that the quality improves with storage duration [46]. Given its extended storage requirements–typically over three years before use as a medicinal herb–CP faces potential mycotoxin contamination risks. However, multi-mycotoxin contamination in CP has not been extensively documented.

Surprisingly, CP showed the lowest contamination risk among the four matrices studied. Only five mycotoxins were detected in 131 samples, with a detection rate of 38.2%, primarily from *Penicillium* species (Figure 4) (Table A6). Consistent with previous research, MPA showed the highest detection rate (35.9%), mainly produced by *Penicillium* [47], confirming citrus fruits’ susceptibility to Penicillium contamination [48]. Similar to LR, BEA-positive samples showed concurrent ENN detection, following the same contamination pattern of ENN B > ENN B1 > ENN A1, suggesting possible contamination by the same Fusarium species. However, our findings differ from previous studies on fresh citrus peel fungal communities, which identified *Erythrobasidium*, *Penicillium*, *Aspergillus*, *Rhodotorula*, and *Mycosphaerella* as the dominant genera, with the rare detection of Fusarium [49]. The low levels of BEA and ENNs in CP suggest initial field contamination by multiple fungi including *Fusarium*, *Penicillium*, and *Aspergillus*, with *Fusarium* gradually being replaced by other dominant fungi.

#### 2.3.4. Coix Seed (CS)

CS is a widely used medicinal and edible grain that has gained popularity as a health food, especially among women, for its properties in eliminating dampness and reducing swelling. It is increasingly consumed as a daily beverage alternative to coffee.

Among 47 samples, 27 mycotoxins were detected, with a total detection rate of 89.4% (Table A7). The analysis of mycotoxin-producing fungi revealed that CS was most susceptible to *Aspergillus* and *Fusarium* contamination (Figure 4). *Fusarium* mycotoxins showed the highest detection rates, with FBs (2.9–430.7 μg/kg) at 74.4% and ZEN (4.1–206.9 μg/kg) at 59.6%. Additionally, AFs showed a significant detection rate of 27.7%, validating the necessity of aflatoxin and zearalenone limits in coix seeds as specified in the Ch.P.

Consistent with previous literature reports, CS showed significant multi-mycotoxin contamination, including both parent mycotoxins and their modified forms [12]. The most severely contaminated sample contained 17 different mycotoxins, with over 50% of samples containing at least four mycotoxins (Figure 5). Samples with high levels of parent mycotoxins often showed a concurrent detection of their modified forms. For example, sample CS3 contained ZEN along with ZAN, α-ZEL, β-ZEL, and α-ZAL, while sample CS6 showed both DON and 3-Ac-DON. Modified mycotoxins showed lower detection rates and contamination levels compared to their parent compounds.

Interestingly, ENNs, which were common contaminants in the other three matrices and are produced by *Fusarium*, were not detected in CS samples. This might be related to differences in the dominant fungal species colonizing CS, suggesting possible competitive relationships among fungi.

### 2.4. Health Risk Assessment

A health risk assessment was performed according to the guidelines established by the International Agency for Research on Cancer (IARC) and the Joint FAO/WHO Expert Committee on Food Additives (JECFA), evaluating mycotoxins that showed detection frequencies above 20% in the studied matrices.

#### 2.4.1. Estimation of Exposure

The exposure to mycotoxins was calculated using the average contamination levels from 260 batches of four matrices, average body weight, and daily intake doses. Daily intake doses were based on the maximum recommended dosages specified in the 2020 edition of the Ch.P, with maximum daily intakes set at 15 g for LS, 10 g for LR, 10 g for CP, and 30 g for CS. The average body weight of 64.4 kg was derived from the “Report on Nutrition and Chronic Diseases of Chinese Residents (2020)”, accounting for male-to-female population ratios. Mycotoxin exposure was calculated using the following formula, with detailed exposure levels presented in Table 3.

Exposure = (C × IR)/BW

C represents the average mycotoxin contamination level in medicinal materials (ng/g); IR represents the daily intake rate (g·day^−1^); and BW represents the average body weight (kg). Following the principles for handling non-detect data from the WHO Global Environment Monitoring System/Food Contamination Monitoring and Assessment Programme (GEMS/Food) Second Workshop on “Reliable Evaluation of Low-Level Contamination of Food” and the standards proposed in the European Commission’s Scientific Cooperation Task 3.2.10 (SCOOP) [50], the contamination level was calculated as the mean of all samples, with non-detect samples assigned a value of 1/2 LOD.

#### 2.4.2. Risk Assessment of Mycotoxins Based on Margin of Exposure Margin of Exposure (MOE)

For non-threshold carcinogenic chemical hazards such as aflatoxins, risk assessment was conducted using the MOE approach, based on the Benchmark Dose Lower confidence limit of 10% extra risk (BMDL_10_) parameters published by EFSA (see Table 3) [51]. An MOE value greater than 10,000 indicates an acceptable health risk, while values below this threshold suggest potential health concerns.

MOE = BMDL_10_/Exposure

**Figure 9:**
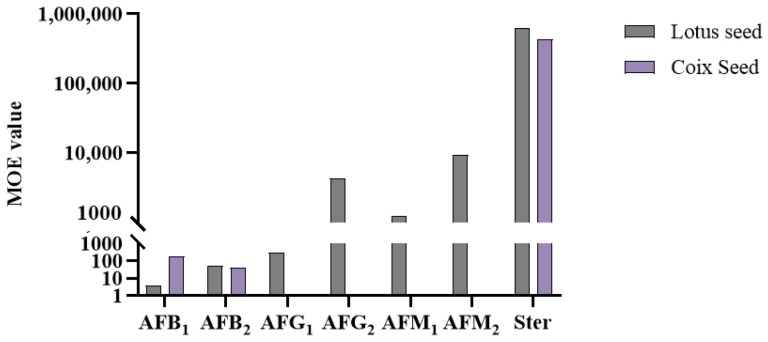
MOE values from lotus seed and coix seed consumption for Chinese people.

MOE values were calculated for seven mycotoxins (AFB_1_, AFB_2_, AFG_1_, AFG_2_, AFM_1_, AFM_2_, and Ster), as shown in Figure 9. CP and LR were excluded from the analysis due to the non-detection of the relevant mycotoxins. The analysis revealed that Ster posed minimal risks in both LS and CS for its lower toxicity. However, the MOE values for the remaining six aflatoxins were all below 10,000, indicating potential health concerns. While AFM_2_ showed values approaching 10,000, the actual risk might be higher considering that LS, as both medicinal and food items, may be consumed in larger quantities than the calculated dose. Additionally, children with lower body weights may face elevated risks. These findings highlight the necessity for monitoring aflatoxin risks in LS and CS.

#### 2.4.3. Risk Assessment of Mycotoxins Based on Hazard Index (HI)

For threshold hazardous compounds such as DON and ZEN, a risk assessment was conducted using the HI method, based on the Provisional Maximum Tolerable Daily Intake (PMTDI) [52]. The HI is calculated as the sum of Hazard Quotients (HQ) for individual chemical compounds. An HI value less than 1 indicates an acceptable exposure risk level, while values exceeding 1 suggest potential adverse effects on human health. For certain mycotoxins with insufficient toxicological data and no official PMTDI values, a reference value of 2000 ng·kg^−1^ b.w.·day^−1^ was adopted (including BEA, CPA, ENNs, and MPA), based on fumonisin toxicity data.

HQ = Exposure/PMTDI

HI = ∑HQ

**Figure 10:**
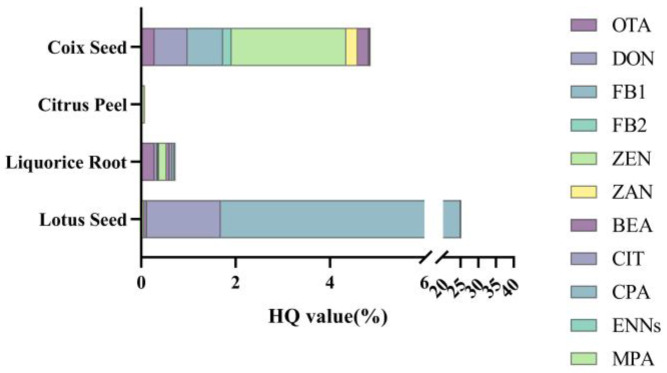
HQ values from lotus seed and coix seed consumption for Chinese people.

As shown in Figure 10, the overall HI values for all four matrices were relatively low, with ZEN and CIT being the primary risk contributors. Among these, CPA was the largest contributor to the HI in LS, with a value of 0.23. Although CS showed low overall risk, the high detection rate of fumonisins (74.4%) suggests potential exposure risks, indicating the need for expanded data collection to comprehensively evaluate the necessity of including these compounds in regulatory standards.

## 3. Conclusions

This study systematically revealed the mycotoxin contamination characteristics and potential risks in four dual-use medicinal and edible plants through contamination analysis and health risk assessment.

Contamination analysis demonstrated that different types of plants exhibited unique contamination profiles: seed-type plants (LS and CS) were susceptible to *Aspergillus* and *Fusarium* contamination, with detection rates of 53.3% and 22.2%, respectively. Despite low detection rates, the MOE values of six aflatoxins remained below 10,000 due to their high toxicity, indicating potential health risks. Peel-type materials (citrus peel) were primarily contaminated with MPA produced by *Penicillium* (35.9% detection rate), while root-type materials (licorice) were mainly affected by *Fusarium* species (52.9% detection rate).

Significant differences were observed in contamination levels between samples of different physical states, with fragmented samples showing more severe mycotoxin contamination. Powdered LS showed a 52.9% higher detection rate of AFB_1_ compared to whole seeds, while sliced LR demonstrated a 41.2% higher detection rate of FB_1_ than intact samples. Three highly positive lotus samples, including moldy samples, discolored samples, and farm-cultivated samples, although limited in number, suggested the importance of timely drying and standardized sourcing in preventing mycotoxin contamination. GR samples collected between 2015 and 2020 and stored under cool conditions showed no significant differences in mycotoxin contamination.

Furthermore, highly contaminated samples revealed the co-occurrence of metabolically related toxins, such as the simultaneous detection of AFM_1_ (2.1 μg/kg) and AFM_2_ (1.8 μg/kg) in lotus seeds, providing new directions for studies on the metabolic mechanisms of mycotoxins in plants. Interestingly, the CP samples included in this study showed very low exposure risk (HI = 0.07%), possibly attributed to their specific processing techniques (e.g., low-temperature drying) or natural antimicrobial components.

The multi-toxin detection method established in this study can be extended to other plants with similar compositions, providing important technical support and data references for establishing scientific quality control systems. Based on our findings, we propose the following recommendations for industrial applications: (1) Emphasize supplier qualification verification during raw material procurement and strictly control moisture content; (2) Process and store materials in a non-fragmented state when possible and use sealed packaging after drying to prevent moisture absorption; (3) Maintain storage temperatures below 20 °C during storage and transportation and utilize dehumidification equipment when possible.

## 4. Materials and Methods

### 4.1. Sample Collection

A total of 260 samples of four edible and medicinal plants were collected, including LR (77 batches), LS (29 batches), CS (47 batches), and CP (131 batches). The majority of samples were obtained through national or local quality surveillance programs from various provinces in China, with a few collected directly from local farmers. All samples were authenticated by Chief Pharmacist Yang Xinhua from the Traditional Chinese Medicine/Natural Medicine and Health Food Institute, Shanghai Institute of Food and Drug Control. For each matrix, 200 g of sample was collected, ground into fine powder, passed through a 50-mesh sieve, and stored at −20 °C.

### 4.2. Chemicals and Reagents

Acetonitrile and methanol were purchased from Merck (Darmstadt, Germany). LC-MS grade acetic acid and formic acid were supplied by Fisher Scientific (Somerville, USA). Ammonium formate and ammonium acetate were obtained from Sigma Aldrich (Zwijndrecht, The Netherlands). Analytically pure acetic acid, formic acid, ammonium formate, and ammonium acetate were purchased from Merck (Darmstadt, Germany). Deionized water was obtained using a Milli-Q Gradient Water System (Millipore, Bedford, MA, USA).

Anhydrous magnesium sulfate, sodium chloride, trisodium citrate dihydrate, disodium citrate hydrate, anhydrous sodium acetate, dispersed solid-phase extraction (d-SPE) sorbent octadecylsilane (C18), primary secondary amine (PSA), silica gel (Si), and propane sulfonic acid (PRS) were obtained from Bonna-Agela Technologies (Tianjin, China). Solid reagent anhydrous sodium acetate prepared for buffer solution was obtained from Sinopharm Chemical Reagent Co. Ltd. (Shanghai, China). All other reagents were of analytical grade.

Solid standards or stock solutions were collected from various sources (Table A8) and the information on the 73 mycotoxins’ standards is listed in Table 1. The declared purities of all standards ranged from 92.87% to 99.9%.

### 4.3. Preparation of Standard Solution

Stock solutions of mycotoxins were prepared in acetonitrile at concentrations ranging from 10 to 250 μg/mL and stored in brown glass vials at −20 °C, respectively. Based on their mass spectrometric response intensities, the 73 mycotoxins were divided into three groups. Group 1 (G1) included the following mycotoxins: 7-dechloro griseofulvin, aflatoxin B1, B2, G1, G2, and M1, agroclavine, anisomycin, beauvericin, diacetoxyscirpenol, dihydrolysergamide, enniatin A, enniatin A1, enniatin B, enniatin B1, ergocornine, ergocorninine, ergocristine, ergocristinine, ergocryptine, ergocryptinine, ergosine, griseofulvin, lysergamide, meleagrin, mycophenolic acid, ochratoxin A, ochratoxin B, ochratoxin C, oxaline, puromycin, roquefortine C, and sterigmatocystin. Group 2 (G2) included 15-acetoxyscirpenol, aflatoxin M2, aflatoxin P1, apicidin, chaetocin, citrinin, cyclopiazonic acid, equisetin, fumonisin B1, fumonisin B2, fumonisin B3, gliotoxin, monocerin, neosolaniol, o-methylsterigmatocystin, pseurotin A, secalonic acid D, T-2 toxin, α-zearalanol, α-zearalenol, β-zearalanol, β-zearalenol, and zearalenone. Group 3 (G3) included 15-acetyldeoxynivalenol, 3-acetyldeoxynivalenol, chetomin, citreoviridin, deepoxy-deoxynivalenol, deoxynivalenol, fumagillin, fusarenon X, HT-2 toxin, Ostreogrycin A, T-2-triol, tentoxin, wortmannin, patulin, alternariol-methylether, and alternariol.

Mixed standard stock solutions were prepared by combining individual stock solutions from each group to achieve the following concentrations: 100 ppb for G1, 500 ppb for G2, and 2500 ppb for G3. Working standard solutions at various concentration levels were subsequently prepared by appropriate dilution with suitable solvents as required by the analytical method.

### 4.4. Sample Preparation

An accurately weighed 2.0 g portion of homogenized sample was transferred into a 50 mL polypropylene centrifuge tube. Extraction was carried out with 20 mL of acetonitrile–water–acetic acid (80:19:1, *v*/*v*/*v*) using an orbital shaker (IKA, Guangzhou, China) for 90 min, followed by centrifugation (Eppendorf, Hamburg, Germany) at 3900 rpm for 5 min. Subsequently, 10 mL of the supernatant was transferred and combined with 10 mL of 5% formic acid solution. A QuEChERS salt mixture (sodium chloride, anhydrous magnesium sulfate, sodium citrate, and sodium citrate sesquihydrate; 1 g:4 g:1 g:0.5 g) was added immediately, followed by high-speed vortexing for 5 min (SPEX, New York, NY, USA) and centrifugation at 3900 rpm for 5 min. For further purification, 6.0 mL of the supernatant was subjected to liquid–liquid partitioning with 12 mL cyclohexane, followed by centrifugation at 3900 rpm. The lower phase (4 mL) was collected and concentrated to near dryness under a gentle nitrogen stream at 40 °C. The residue was reconstituted in 0.5 mL acetonitrile and diluted to 2 mL with water. The final extract was filtered through a 0.22 μm PTFE membrane filter (Agilent, Shanghai, China) prior to UHPLC-MS/MS analysis, with an injection volume of 5 μL.

### 4.5. UHPLC-MS/MS Analysis

Chromatographic separation was performed on a 1290 UHPLC system equipped with a quaternary solvent delivery system, degasser, autosampler, and column thermostat, coupled to a 5500 triple quadrupole mass spectrometer (AB SCIEX, Framingham, MA, USA) with an electrospray ionization (ESI) source operating in both positive and negative modes.

Chromatographic separation of the 73 mycotoxins was achieved on a Poroshell EC-C18 column (150 × 3.0 mm, 2.7 μm) (Agilent, Wilmington, DE, USA) at a flow rate of 450 μL/min. For the 64 mycotoxins analyzed in the positive mode (ESI^+^), mobile phase A consisted of 0.4% formic acid and 2.0 mM ammonium formate in water and mobile phase B consisted of 0.4% formic acid and 2.0 mM ammonium formate in methanol. The gradient program was 0–2 min, 20% B; 2–6 min, 20–50% B; 6–11 min, 50–55% B; 11–15 min, 55–100% B; 15–19 min, 100% B; 19–21 min, 100–20% B; and 21–25 min, 100% B. For the remaining mycotoxins analyzed in negative mode (ESI^−^), mobile phase A was water and mobile phase B was acetonitrile, with the following gradient: 0–2 min, 10% B; 2–8 min, 10–50% B; 8–13 min, 50–60% B; 13–15 min, 60–100% B; 15–16 min, 100% B; 16–18 min, 100–10% B; and 18–20 min, 10% B. The injection volume was 1.0 μL, the column temperature was maintained at 35 °C, and the sample tray temperature was set at 15 °C to enhance sample stability.

Mass spectrometric detection was performed under the following conditions: for the positive mode, the ion spray voltage was 5.5 kV, curtain gas was 30 psi, ion source gas 1 and gas 2 were both 50 psi, and source temperature was 450 °C; for the negative mode, the ion spray voltage was 4.5 kV, curtain gas was 30 psi, ion source gas 1 and gas 2 were both 50 psi, and source temperature was 400 °C.

A multiple reaction monitoring (MRM) mode was employed, with at least one precursor ion and two product ions monitored for each mycotoxin. The two most intense product ions free from matrix interference were selected for quantification and qualification, respectively. Declustering potentials (DPs) and collision energies (CEs) were optimized individually using standard solutions for each analyte.

Data acquisition and processing were performed using Analyst 1.5.1 and MultiQuant™ 2.1.1 software (AB SCIEX). Detailed information regarding the retention times (RTs), monitored precursor and product ions, and optimized DPs and CEs for each mycotoxin is presented in Table 1.

### 4.6. Statistical Analysis

All statistical analyses were performed using R software (version 4.2.0; R Core Team, 2023). Due to the non-normal distribution of data and presence of numerous null values, non-parametric analyses were conducted using the “stats” package. The wilcox.test() function was employed for Mann–Whitney U tests to evaluate differences in mycotoxin contamination between different forms of samples, while the kruskal.test() function was used for Kruskal–Wallis tests to analyze differences among different harvest years. Data visualization was accomplished using the “ggplot2” package. Statistical significance was defined as *p* < 0.05.

## Figures and Tables

**Table 1:** Chemical Information of 73 mycotoxins.

No.	Mycotoxin	Abbreviation	Formula
1	15-Acetoxyscirpenol	15-Asp	C_17_H_24_O_6_
2	15-Acetyldeoxynivalenol	15-ADON	C_17_H_22_O_7_
3	3-Acetyldeoxynivalenol	3-ADON	C_17_H_22_O_7_
4	7-Dechloro Griseofulvin	7-D-G	C_17_H_18_O_6_
5	Aflatoxin B_1_	AFB_1_	C_17_H_12_O_6_
6	Aflatoxin B_2_	AFB_2_	C_17_H_14_O_6_
7	Aflatoxin G_1_	AFG_1_	C_17_H_12_O_7_
8	Aflatoxin G_2_	AFG_2_	C_17_H_14_O_7_
9	Aflatoxin M_1_	AFM_1_	C_17_H_12_O_7_
10	Aflatoxin M_2_	AFM_2_	C_17_H_14_O_7_
11	Aflatoxin P_1_	AFP_1_	C_16_H_10_O_6_
12	Agroclavine	Agro	C_16_H_18_N_2_
13	Anisomycin	Anis	C_14_H_19_NO_4_
14	Apicidin	Apici	C_34_H_50_N_5_O_6_
15	Beauvericin	BEA	C_45_H_57_N_3_O_9_
16	Chaetocin	Chae	C_30_H_28_N_6_O_6_S_4_
17	Chetomin	Che	C_31_H_30_N_6_O_6_S_4_
18	Citrinin	CIT	C_13_H_14_O_5_
19	Citreoviridin	CVD	C_23_H_30_O_6_
20	Cyclopiazonic acid	CPA	C_20_H_20_N_2_O_3_
21	Diacetoxyscirpenol	DAS	C_19_H_26_O_7_
22	Dihydrolysergamide	DiLy	C_16_H_19_N_3O_
23	Deepoxy-deoxynivalenol	DOM	C_15_H_20_O_5_
24	Deoxynivalenol	DON	C_15_H_20_O_6_
25	Enniatin A	ENN A	C_36_H_63_N_3_O_9_
26	Enniatin A1	ENN A1	C_35_H_61_N_3_O_9_
27	Enniatin B	ENN B	C_33_H_57_N_3_O_9_
28	Enniatin B1	ENN B1	C_34_H_59_N_3_O_9_
29	Equisetin	Equi	C_22_H_31_NO_4_
30	Ergocornine	EGCN	C_31_H_39_N_5_O_5_
31	Ergocorninine	EGCNN	C_31_H_39_N_5_O_5_
32	Ergocristine	EGST	C_35_H_39_N_5_O_5_
33	Ergocristinine	EGSTN	C_35_H_39_N_5_O_5_
34	Ergocryptine	EGPT	C_32_H_41_N_5_O_5_
35	Ergocryptinine	EGPTN	C_32_H_41_N_5_O_5_
36	Ergosine	EGSN	C_30_H_37_N_5_O_5_
37	Fumonisin B1	FB_1_	C_34_H_59_NO_15_
38	Fumonisin B2	FB_2_	C_34_H_59_NO_14_
39	Fumonisin B3	FB_3_	C_34_H_59_NO_14_
40	Fumagillin	Fum	C_26_H_34_O_7_
41	Fusarenon X	FuX	C_17_H_22_O_8_
42	Gliotoxin	Glio	C_13_H_14_N_2_O_4_S_2_
43	Griseofulvin	Grise	C_17_H_17_ClO_6_
44	HT-2 toxin	HT-2	C_22_H_32_O_8_
45	Lysergamide	Lyser	C_16_H_17_N_3_O
46	Meleagrin	Melea	C_23_H_23_N_5_O_4_
47	Monocerin	MONO	C_16_H_20_O_6_
48	Mycophenolic Acid	MPA	C_17_H_20_O_6_
49	Neosolaniol	NEO	C_19_H_26_O_8_
50	O-methylsterigmatocystin	O-m-Ster	C_19_H_14_O_6_
51	Ostreogrycin A	Ostre A	C_28_H_35_N_3_O_7_
52	Ochratoxin A	OTA	C_20_H_18_ClNO_6_
53	Ochratoxin B	OTB	C_20_H_19_NO_6_
54	Ochratoxin C	OTC	C_22_H_22_ClNO_6_
55	Oxaline	Oxa	C_24_H_25_N_5_O_4_
56	Pseurotin A	Pse A	C_22_H_25_NO_8_
57	Puromycin	Puro	C_22_H_29_N_7_O_5_
58	Roquefortine C	Rq C	C_22_H_23_N_5_O_2_
59	Secalonic acid D	Secal Acid D	C_32_H_30_O_14_
60	Sterigmatocystin	Ster	C_18_H_12_O_6_
61	T-2-triol	T2-tri	C_20_H_30_O_7_
62	T-2 toxin	T-2	C_24_H_34_O_9_
63	Tentoxin	Ten	C_22_H_30_N_4_O_4_
64	Wortmannin	Wor-man	C_23_H_24_O_8_
65	α-zearalanol	α-ZAL	C_18_H_26_O_5_
66	α-zearalenol	α-ZEL	C_18_H_24_O_5_
67	β-zearalanol	β-ZAL	C_18_H_26_O_5_
68	β-zearalenol	β-ZEL	C_18_H_24_O_5_
69	Patulin	PAT	C_7_H_6_O_4_
70	Zearalanone	ZAN	C_18_H_24_O_5_
71	Zearalenone	ZEN	C_18_H_22_O_5_
72	Alternariol-methylether	AME	C_15_H_12_O_5_
73	Alternariol	AOH	C_14_H_10_O_5_

**Table 2:** Optimized MS/MS parameters for the analytes studied.

Mycotoxin	MS	RT (min)	Precursor Ion	DP (V)	Product Ion									
					1		2		3		4		5	
					Ion	CE (V)	Ion	CE (V)	Ion	CE (V)	Ion	CE (V)	Ion	CE (V)
15-Asp	[M+NH_4_]^+^	8.6	342.0	10	265.3	13	307.2	12						
15-ADON	[M+H]^+^	7.6	339.1	130	137.1	23	261.1	16	321.1	19	261.0	16	304.4	19
3-ADON	[M+H]^+^	7.6	339.1	130	231.1	17	203.1	20	304.1	19	181.2	24		
7-D-G	[M+H]^+^	11.8	319.1	22	181.2	24	251.1	24						
AFB_1_	[M+H]^+^	10.5	313.0	200	241.0	50	285.1	33	269.0	42	269.0	42	214.0	40
AFB_2_	[M+H]^+^	9.7	315.1	200	287.1	35	259.1	40	243.1	52	203.0	50	271.0	46
AFG_1_	[M+H]^+^	9.0	329.1	200	243.1	35	215.1	45	311.1	30	283.0	33		
AFG_2_	[M+H]^+^	8.4	331.1	200	313.1	33	245.1	40	217.1	47	257.0	42	189.0	55
AFM_1_	[M+H]^+^	8.5	329.1	60	273.1	33	259.2	33						
AFM_2_	[M+H]^+^	7.9	331.1	140	273.1	47	259.3	47	285.0	47				
AFP_1_	[M+H]^+^	8.9	299.0	180	271.1	33	215.1	39	187.1	43	201.1	39		
Agro	[M+H]^+^	7.2	239.1	20	183.1	33	168.2	36	198.2	35	207.9	35		
Anis	[M+H]^+^	6.2	266.2	20	206.1	22	121.0	35						
Apici	[M+H]^+^	15.6	624.3	190	464.3	25	592.3	21						
BEA	[M+NH_4_]^+^	16.4	801.4	10	244.2	40	262.0	40						
Chae	[M+H]^+^	15.0	697.1	90	348.0	26	284.3	36						
Che	[M+H]^+^	15.3	711.2	150	644.9	17	647.2	17	348.0	28				
CIT	[M+H]^+^	12.3	251.2	90	233.1	25	205.1	35						
CVD	[M+H]^+^	15.1	403.2	220	139.0	30	297.1	20						
CPA	[M+H]^+^	15.7	337.2	140	182.0	25	196.0	47	319.1	35				
DAS	[M+H]^+^	10.4	384.2	40	307.3	15	229.1	20	247.1	19				
DiLy	[M+H]^+^	4.1	270.1	40	225.1	30	168.1	30						
DOM	[M+H]^+^	6.3	281.1	130	215.1	18	233.1	16						
DON	[M+H]^+^	4.8	297.1	120	249.1	17	231.1	18						
ENN A	[M+NH_4_]^+^	16.6	699.5	5	210.2	41	228.2	42						
ENN A_1_	[M+NH_4_]^+^	16.5	685.5	5	210.2	38	228.2	39						
ENN B	[M+NH_4_]^+^	16.3	657.3	5	196.1	39	214.2	41						
ENN B_1_	[M+NH_4_]^+^	16.4	671.5	5	196.1	40	210.1	38						
Equi	[M+H]^+^	16.7	374.1	21	175.1	23	200.0	23						
EGCN	[M+H]^+^	10.2	562.3	10	268.2	36	305.2	34						
EGCNN	[M+H]^+^	11.8	562.3	40	544.1	26	277.2	38						
EGST	[M+H]^+^	12.5	610.3	40	268.1	40	223.1	40						
EGSTN	[M+H]^+^	13.2	610.3	5	592.3	22	268.2	33						
EGPT	[M+H]^+^	12.0	576.4	20	223.0	40	304.9	40						
EGPTN	[M+H]^+^	13.0	576.4	30	558.2	21	305.3	38	223.0	49				
EGSN	[M+H]^+^	9.7	548.2	30	530.0	20	268.1	20	223.2	20	277.4	20		
FB_1_	[M+H]^+^	14.3	722.4	150	334.3	55	352.3	49						
FB_2_	[M+H]^+^	15.0	706.4	150	336.3	49	318.3	52						
FB_3_	[M+H]^+^	14.7	706.6	140	336.3	47	688.5	47						
Fum	[M+H]^+^	15.7	459.2	140	131.0	42	177.0	25						
FuX	[M+H]^+^	6.2	355.1	60	247.0	30	229.1	20						
Glio	[M+H]^+^	10.7	327.1	5	263.2	15	244.8	23						
Grise	[M+H]^+^	13.6	353.1	50	215.0	25	163.0	25	285.1	25				
HT-2	[M+H]^+^	13.7	442.2	50	263.1	17	215.1	19						
Lyser	[M+H]^+^	4.0	268.4	64	208.1	31	223.2	27						
Melea	[M+H]^+^	10.7	434.2	5	403.3	22	334.1	30	289.1	40				
Mono	[M+H]^+^	13.8	309.1	47	223.1z	22	291.1	21	273.3	21				
MPA	[M+H]+	14.5	321.3	47	207.1	25	302.9	12						
NEO	[M+NH_4_]^+^	6.4	400.1	10	185.0	26	215.0	25						
O-m-Ster	[M+H]^+^	14.6	339.0	10	278.1	42	295.0	38	306.1	38	324.1	32		
Ostre A	[M+NH_4_]^+^	14.0	543.3	140	508.2	24	355.3	32	337.3	36				
OTA	[M+H]^+^	15.2	404.1	100	239.0	34	102.1	93	358.0	16	221.0	43	193.0	50
OTB	[M+H]^+^	14.5	370.0	60	205.2	47	324.4	18	103.0	70				
OTC	[M+H]^+^	15.9	432.1	10	239.1	37	358.0	23	386.0	16	341.1	30		
Oxa	[M+H]^+^	10.8	448.1	5	348.1	32	332.1	38						
Pse A	[M+H]^+^	9.9	432.4	20	316.2	14	348.1	7						
Puro	[M+H]^+^	7.4	472.3	10	309.3	27	164.0	36	150.1	36				
Rq C	[M+H]^+^	13.7	390.1	40	193.2	35	322.2	35						
Secal A	[M+H]^+^	15.8	639.2	180	561.2	28	589.0	28	579.2	30				
Ster	[M+H]^+^	15.2	325.1	40	281.1	48	310.1	35	254.0	47				
T2-triol	[M+NH_4_]^+^	6.4	400.2	40	214.9	20	233.3	12	281.3	12	263.0	15		
T-2	[M+NH_4_]^+^	14.5	484.3	50	305.1	19	245.1	18	215.1	26				
Ten	[M+H]^+^	14.0	415.2	175	312.3	29	256.1	45						
Wor-man	[M+H]^+^	15.5	429.5	45	355.1	14	295.2	31						
α-ZAL	[M−H]^−^	10.3	321.1	−180	259.1	−30	303.1	−28	161.0	−37				
α-ZEL	[M−H]^−^	10.5	319.1	−145	173.8	−34	160.0	−42	130.0	−42	188.1	−36		
β-ZAL	[M−H]^−^	9.6	321.1	−138	303.1	−31	259.1	−32	160.9	−38	189.1	−37		
β-ZEL	[M−H]^−^	9.7	319.1	−134	187.9	−35	160.1	−40	174.0	−33				
PAT	[M−H]^−^	4.6	153.0	−5	109.0	−10	81.0	−16	83.0	−19	125.0	−13		
ZAN	[M−H]^−^	12.3	319.1	−148	205.3	−29	161.1	−37	137.1	−36	177.1	−36	187.1	−30
ZEN	[M−H]^−^	12.4	317.1	−90	187.0	−36	174.9	−32	131.2	−38				
AME	[M−H]^−^	12.5	271.0	−160	255.9	−30	228.0	−38	213.0	−48	183.1	−54		
AOH	[M−H]^−^	9.5	257.0	−180	213.0	−31	215.0	−32	147.0	−42	212.0	−38		

**Table 3:** Exposure of detected mycotoxins in four matrices for Chinese people.

Mycotoxin	Exposure (ng·kg^−1^ b.w.day^−1^)				PMTDI ^a^ (ng·kg^−1^ b.w.day^−1^)	BDML_10_^b^ (ng·kg^−1^ b.w.day^−1^)
	LS	LR	CP	CS		
AFB_1_	103.70	0	0	0.23	/	400
AFB_2_	7.55	0	0	0.09	/	400
AFG_1_	1.40	0	0	0	/	400
AFG_2_	0.09	0	0	0	/	400
AFM_1_	3.19	0	0	0	/	4000
AFM_2_	0.54	0	0	0	/	4000
Ster	0.26	0	0	0.05	/	160,000
OTA	0	0.06	0	0.09	16	/
DON	0	0.99	0	7.45	1000	/
FB_1_	0	0.40	0	15.00	2000	/
FB_2_	0	0.28	0	3.73	2000	/
FB_3_	0	0	0	0.79	2000	/
ZEN	0.42	0.90	0	12.20	500	/
ZAN	0	0	0	1.30	500	/
BEA	1.23	1.38	0.14	4.84	2000	/
CIT	3.17	0.20	0	0	200	/
CPA	468.61	0	0	0.42	2000	/
ENNs	1.12	0.81	0.31	0	2000	/
MPA	0.93	0.47	1.01	0.28	2000	/

^a^ Provisional maximum tolerable daily intake (PMTDI) established by the Joint FAO/WHO Expert Committee on Food Additives (JECFA). ^b^ BMDL_10_ was the benchmark dose lower confidence limit of 10% extra risk.

## Data Availability

The original contributions presented in this study are included in the article. Further inquiries can be directed to the corresponding author(s).

## Appendix A

**Table A1:** Overview of the extraction recovery (R), repeatability (RSD), limit of detection (LOD), and limit of quantification (LOQ) for each mycotoxin in LS.

Mycotoxin	Spiked Levels (μg/kg)						LOD (μg/kg)	LOQ (μg/kg)	Linear Range (μg/kg)	Coefficient (r)
	Level 1		Level 2		Level 3					
	R (%)	RSD (%)	R (%)	RSD (%)	R (%)	RSD (%)				
15-Asp	88.8	3.1	86.4	2.3	83.1	3.5	1.25	2.5	2~1000	0.99973
15-ADON	81.9	5.2	82.2	4.2	79.8	4.1	6.25	12.5	10~5000	0.99990
3-ADON	90.8	2.7	89.1	2.2	88.0	3.5	6.25	12.5	10~5000	0.99973
7-D-G	94.9	1.0	97.0	2.2	94.9	3.7	0.25	0.5	0.4~200	0.99959
AFB_1_	91.5	2.0	92.3	2.8	86.1	3.4	0.25	0.5	0.4~200	0.99967
AFB_2_	92.4	1.4	92.5	2.2	88.3	2.2	0.25	0.5	0.4~200	0.99995
AFG_1_	91.1	1.4	90.5	4.2	83.8	0.9	0.25	0.5	0.4~200	0.99993
AFG_2_	87.2	1.9	83.4	3.8	82.2	4.8	0.25	0.5	0.4~200	0.99985
AFM_1_	86.8	3.3	89.1	3.0	87.4	2.2	0.25	0.5	0.4~200	0.99925
AFM_2_	89.2	2.1	89.8	3.9	85.6	3.6	1.25	2.5	2~1000	0.99929
AFP_1_	90.0	2.4	82.1	2.0	84.9	7.5	1.25	2.5	2~1000	0.99993
Agro	82.8	2.5	87.6	3.9	86.2	2.4	0.25	0.5	0.4~200	0.99964
Anis	96.4	2.7	102.5	2.7	103.3	1.9	0.25	0.5	0.4~200	0.99936
Apici	80.4	3.2	81.9	2.8	79.2	3.5	1.25	2.5	2~1000	0.99988
BEA	63.5	5.9	69.5	6.7	69.4	11.4	0.25	0.5	0.4~200	0.99985
Chae	82.9	9.6	91.3	6.8	88.9	8.4	6.25	12.5	10~5000	0.99928
Che	85.9	9.0	85.7	3.7	81.3	3.9	1.25	2.5	2~1000	0.99986
CIT	75.6	2.6	72.9	3.0	73.4	4.4	1.25	2.5	2~1000	0.99991
CVD	81.3	4.7	84.2	2.8	85.0	4.2	6.25	12.5	10~5000	0.99987
CPA	69.1	5.0	73.1	3.1	77.1	8.9	1.25	2.5	2~1000	0.99964
DAS	84.3	4.6	88.2	3.0	89.9	3.4	0.25	0.5	0.4~200	0.99928
DiLy	72.7	6.5	85.0	1.6	88.2	3.7	0.25	0.5	0.4~200	0.99942
DOM	92.5	2.4	89.6	8.2	87.0	6.5	6.25	12.5	10~5000	0.99977
DON	89.0	1.4	89.3	3.6	89.3	2.5	6.25	12.5	10~5000	0.99978
ENN A	76.0	3.5	71.8	5.0	72.1	8.7	0.25	0.5	0.4~200	0.99985
ENN A_1_	73.6	4.6	78.1	3.4	78.0	6.3	0.25	0.5	0.4~200	0.99984
ENN B	80.1	3.5	82.3	2.4	83.1	5.1	0.25	0.5	0.4~200	0.99964
ENN B_1_	81.3	2.0	83.9	3.2	88.0	5.0	0.25	0.5	0.4~200	0.99990
Equi	71.4	2.0	71.5	1.7	77.2	6.9	1.25	2.5	2~1000	0.99957
EGCN	82.0	2.8	87.9	1.8	85.0	3.0	0.25	0.5	0.4~200	0.99957
EGCNN	93.5	4.0	100.0	3.2	91.8	3.0	0.25	0.5	0.4~200	0.99984
EGST	101.4	1.9	103.7	3.9	103.2	4.9	0.25	0.5	0.4~200	0.99977
EGSTN	81.4	2.2	82.7	3.4	87.2	3.0	0.25	0.5	0.4~200	0.99983
EGPT	79.5	1.8	79.7	3.6	79.9	3.9	0.25	0.5	0.4~200	0.99955
EGPTN	90.6	2.1	91.6	2.6	89.6	3.3	0.25	0.5	0.4~200	0.99979
EGSN	116.4	3.1	113.2	3.2	106.1	4.7	0.25	0.5	0.4~200	0.99928
FB_1_	76.4	2.6	77.4	1.7	75.4	3.0	1.25	2.5	2~1000	0.99938
FB_2_	73.4	3.5	76.5	3.5	74.7	5.0	1.25	2.5	2~1000	0.99905
FB_3_	74.3	7.1	81.0	5.4	77.0	5.1	1.25	2.5	2~1000	0.99951
Fum	84.0	12.8	75.6	5.8	79.1	2.6	6.25	12.5	10~5000	0.99933
FuX	87.8	2.3	86.3	5.1	82.7	2.8	6.25	12.5	10~5000	0.99978
Glio	83.9	1.6	82.3	3.9	81.9	3.4	1.25	2.5	2~1000	0.99920
Grise	90.5	1.2	90.0	2.3	85.3	3.6	0.25	0.5	0.4~200	0.99950
HT-2	88.3	1.7	84.9	2.5	82.5	3.4	6.25	12.5	10~5000	0.99978
Lyser	76.3	2.2	80.2	2.0	78.5	3.6	0.25	0.5	0.4~200	0.99959
Melea	83.8	1.9	83.7	3.9	80.6	2.8	0.25	0.5	0.4~200	0.99945
Mono	95.1	0.7	98.8	3.2	95.3	3.5	1.25	2.5	2~1000	0.99997
MPA	114.7	3.0	109.3	2.7	97.9	3.2	0.25	0.5	0.4~200	0.99997
NEO	78.5	3.8	77.5	2.5	76.1	3.9	1.25	2.5	2~1000	0.99979
O-m-Ster	92.3	0.9	99.9	3.3	93.6	3.3	1.25	2.5	2~1000	0.99961
Ostre A	95.5	1.8	103.9	2.3	98.3	2.8	6.25	12.5	10~5000	0.99941
OTA	82.5	4.7	85.2	4.4	83.3	10.2	0.25	0.5	0.4~200	0.99927
OTB	88.2	1.9	95.0	1.7	90.9	4.0	0.25	0.5	0.4~200	0.99972
OTC	80.6	6.2	79.4	2.1	79.8	2.6	0.25	0.5	0.4~200	0.99981
Oxa	83.3	4.7	88.4	4.2	83.6	3.3	0.25	0.5	0.4~200	0.99944
Pse A	95.5	4.2	101.0	3.6	97.7	2.1	1.25	2.5	2~1000	0.99927
Puro	72.8	2.6	78.9	4.9	80.9	5.5	0.25	0.5	0.4~200	0.99959
Rq C	78.8	2.1	74.5	2.5	78.0	3.8	0.25	0.5	0.4~200	0.99966
Secal A	75.4	5.3	88.7	3.6	78.2	6.2	1.25	2.5	2~1000	0.99944
Ster	90.0	6.3	88.7	0.4	81.0	4.1	0.25	0.5	0.4~200	0.99963
T2-triol	83.5	2.8	82.9	2.8	79.3	3.1	6.25	12.5	10~5000	0.99946
T-2	93.9	3.9	94.7	3.7	92.7	3.1	1.25	2.5	2~1000	0.99930
Ten	96.7	2.1	110.5	2.1	109.2	3.1	6.25	12.5	10~5000	0.99913
Wor-man	92.9	3.8	99.6	3.5	93.0	3.7	6.25	12.5	10~5000	0.99999
α-ZAL	88.8	2.7	87.0	2.2	88.0	3.0	1.25	2.5	2~400	0.99939
α-ZEL	89.2	3.5	83.7	1.2	81.8	1.9	1.25	2.5	2~400	0.99955
β-ZAL	94.0	4.2	88.2	2.7	79.6	3.1	1.25	2.5	2~1000	0.99978
β-ZEL	87.2	4.6	84.5	2.8	83.4	2.6	1.25	2.5	2~1000	0.99970
PAT	85.0	2.7	80.5	3.6	78.0	2.4	6.25	12.5	10~5000	0.99946
ZAN	92.6	3.2	83.6	4.4	72.3	2.5	1.25	2.5	2~1000	0.99948
ZEN	94.9	4.8	80.8	2.1	83.5	4.2	1.25	2.5	2~1000	0.99985
AME	91.9	1.7	94.3	2.4	94.5	2.9	6.25	12.5	10~2000	0.99952
AOH	87.0	5.7	77.7	4.7	81.6	3.7	6.25	12.5	10~2000	0.99931

**Table A2:** Overview of the extraction recovery (R), repeatability (RSD), limit of detection (LOD), and limit of quantification (LOQ) for each mycotoxin in GR.

Mycotoxin	Spiked Levels (μg/kg)						LOD (μg/kg)	LOQ (μg/kg)	Linear Range (μg/kg)	Coefficient (r)
	Level 1		Level 2		Level 3					
	R (%)	RSD (%)	R (%)	RSD (%)	R (%)	RSD (%)				
15-Asp	84.6	5.2	79.7	4.5	79.6	4.1	2.5	5.0	2~1000	0.99950
15-ADON	83.6	6.1	83.6	2.4	81.7	6.9	12.5	25.0	10~5000	0.99956
3-ADON	88.5	5.3	81.5	3.2	78.3	3.1	12.5	25.0	10~5000	0.99987
7-D-G	84.0	2.7	81.2	3.2	79.9	2.4	0.5	1.0	0.4~200	0.99916
AFB_1_	95.9	8.0	83.3	9.9	83.6	6.4	0.5	1.0	0.4~200	0.99924
AFB_2_	109.6	7.2	82.5	4.2	79.7	4.5	0.5	1.0	0.4~200	0.99957
AFG_1_	93.7	5.9	75.8	4.5	77.3	7.6	0.5	1.0	0.4~200	0.99946
AFG_2_	87.5	13.2	88.3	9.0	88.8	7.7	0.5	1.0	0.4~200	0.99990
AFM_1_	94.4	6.1	84.3	5.5	87.0	3.5	0.5	1.0	0.4~200	0.99969
AFM_2_	92.2	3.5	80.9	4.3	84.8	4.3	2.5	5.0	2~1000	0.99924
AFP_1_	94.7	7.1	74.0	4.5	72.7	6.1	2.5	5.0	2~1000	0.99959
Agro	78.9	7.3	72.9	6.2	73.3	5.0	0.5	1.0	0.4~200	0.99901
Anis	86.0	4.4	87.4	4.7	87.6	3.3	0.5	1.0	0.4~200	0.99962
Apici	84.9	7.8	75.2	5.7	77.1	6.6	2.5	5.0	2~1000	0.99919
BEA	91.2	5.4	84.3	1.7	81.6	4.3	0.5	1.0	0.4~200	0.99920
Chae	83.3	10.2	75.2	9.4	78.1	6.5	12.5	25.0	10~5000	0.99959
Che	83.2	10.6	79.5	6.4	79.5	8.5	2.5	5.0	2~1000	0.99899
CIT	70.6	3.5	72.4	4.3	74.1	5.3	2.5	5.0	2~1000	0.99963
CVD	86.1	14.2	78.4	7.8	76.5	8.0	12.5	25.0	10~5000	0.99989
CPA	73.6	11.8	67.6	8.8	64.3	4.7	2.5	5.0	2~1000	0.99982
DAS	83.3	3.0	79.8	3.1	78.0	4.1	0.5	1.0	0.4~200	0.99968
DiLy	62.7	2.5	67.4	3.5	65.9	4.9	0.5	1.0	0.4~200	0.99939
DOM	85.2	2.6	82.1	3.2	76.8	2.3	12.5	25.0	10~5000	0.99948
DON	75.4	4.6	76.7	3.4	74.9	3.4	12.5	25.0	10~5000	0.99981
ENN A	86.3	5.0	82.0	2.7	79.3	2.7	0.5	1.0	0.4~200	0.99920
ENN A_1_	87.5	3.6	82.9	3.8	79.3	4.1	0.5	1.0	0.4~200	0.99943
ENN B	92.1	4.9	83.6	3.1	80.3	4.3	0.5	1.0	0.4~200	0.99976
ENN B_1_	85.9	4.1	81.5	4.3	79.9	2.8	0.5	1.0	0.4~200	0.99930
Equi	80.4	7.5	73.1	4.3	69.2	4.1	2.5	5.0	2~1000	0.99892
EGCN	94.2	4.6	73.6	5.2	78.0	4.5	0.5	1.0	0.4~200	0.99928
EGCNN	88.4	3.2	80.6	3.1	77.4	5.2	0.5	1.0	0.4~200	0.99980
EGST	102.8	6.8	86.0	4.1	80.6	3.4	0.5	1.0	0.4~200	0.99946
EGSTN	87.5	4.6	75.1	3.0	71.0	3.2	0.5	1.0	0.4~200	0.99912
EGPT	95.8	6.3	77.5	3.9	70.6	2.9	0.5	1.0	0.4~200	0.99936
EGPTN	85.2	6.5	76.8	4.4	79.8	6.1	0.5	1.0	0.4~200	0.99911
EGSN	94.7	7.6	91.3	4.2	94.0	6.2	0.5	1.0	0.4~200	0.99927
FB_1_	74.5	7.5	76.6	7.9	79.6	8.6	2.5	5.0	2~1000	0.99951
FB_2_	71.7	11.4	77.9	9.4	87.4	11.1	2.5	5.0	2~1000	0.99935
FB_3_	79.2	11.1	86.6	9.2	92.2	4.8	2.5	5.0	2~1000	0.99945
Fum	81.6	7.9	82.2	9.0	105.4	10.3	12.5	25.0	10~5000	0.99945
FuX	84.6	4.8	80.3	2.7	77.0	2.7	12.5	25.0	10~5000	0.99958
Glio	80.7	3.0	82.6	3.4	78.4	4.2	2.5	5.0	2~1000	0.99907
Grise	69.8	7.0	72.2	9.2	80.5	4.4	0.5	1.0	0.4~200	0.99917
HT-2	87.8	4.9	83.7	3.9	82.1	4.2	12.5	25.0	10~5000	0.99949
Lyser	71.3	4.7	67.1	5.0	68.4	3.8	0.5	1.0	0.4~200	0.99998
Melea	87.9	2.2	80.8	3.8	76.1	2.9	0.5	1.0	0.4~200	0.99923
Mono	83.7	3.7	79.4	4.4	76.7	1.6	2.5	5.0	2~1000	0.99972
MPA	82.7	5.4	87.4	6.6	84.2	3.1	0.5	1.0	0.4~200	0.99894
NEO	87.1	1.9	82.8	5.4	77.2	2.5	2.5	5.0	2~1000	0.99909
O-m-Ster	77.9	4.8	70.3	7.4	72.9	14.4	2.5	5.0	2~1000	0.99947
Ostre A	86.7	5.5	75.9	6.1	76.6	9.1	12.5	25.0	10~5000	0.99906
OTA	83.2	6.8	77.7	6.3	80.5	7.3	0.5	1.0	0.4~200	0.99904
OTB	87.2	4.5	85.8	3.4	82.4	2.0	0.5	1.0	0.4~200	0.99940
OTC	98.5	6.1	80.5	4.2	77.5	5.2	0.5	1.0	0.4~200	0.99984
Oxa	85.1	2.8	81.8	4.5	78.8	3.5	0.5	1.0	0.4~200	0.99952
Pse A	86.8	5.4	83.8	4.2	82.4	5.0	2.5	5.0	2~1000	0.99925
Puro	72.2	9.1	69.9	7.7	75.6	4.1	0.5	1.0	0.4~200	0.99917
Rq C	78.3	7.4	73.3	12.7	73.5	14.9	0.5	1.0	0.4~200	0.99989
Secal A	86.7	13.2	78.6	9.4	74.2	7.5	2.5	5.0	2~1000	0.99928
Ster	80.9	10.8	70.8	4.8	70.5	14.6	0.5	1.0	0.4~200	0.99966
T2-triol	93.8	2.6	85.7	4.6	80.3	3.3	12.5	25.0	10~5000	0.99914
T-2	91.6	5.0	86.0	1.6	81.1	3.5	2.5	5.0	2~1000	0.99975
Ten	80.8	7.2	86.0	4.0	88.1	2.1	12.5	25.0	10~5000	0.99995
Wor-man	88.7	2.8	82.9	2.8	80.8	4.1	12.5	25.0	10~5000	0.99984
α-ZAL	85.5	10.2	72.0	6.4	75.2	7.6	2.5	5.0	2~400	0.99973
α-ZEL	83.9	4.3	72.9	5.5	76.8	4.8	2.5	5.0	2~400	0.99908
β-ZAL	76.5	8.0	78.4	10.0	82.8	5.5	2.5	5.0	2~1000	0.99960
β-ZEL	82.5	10.2	94.8	4.1	98.6	13.3	2.5	5.0	2~1000	0.99897
PAT	77.9	8.3	76.7	7.6	78.9	9.8	12.5	25.0	10~5000	0.99957
ZAN	83.4	7.6	78.3	4.6	77.6	2.5	2.5	5.0	2~1000	0.99998
ZEN	90.2	6.9	80.9	3.4	76.5	4.7	2.5	5.0	2~1000	0.99965
AME	98.8	4.1	93.4	2.1	89.7	2.5	12.5	25.0	10~1000	0.99869
AOH	106.9	6.7	93.1	2.5	81.6	4.7	12.5	25.0	10~1000	0.99849

**Table A3:** Overview of the extraction recovery (R), repeatability (RSD), limit of detection (LOD), and limit of quantification (LOQ) for each mycotoxin in CP.

Mycotoxin	Spiked Levels (μg/kg)						LOD (μg/kg)	LOQ (μg/kg)	Linear Range (μg/kg)	Coefficient (r)
	Level 1		Level 2		Level 3					
	R (%)	RSD (%)	R (%)	RSD (%)	R (%)	RSD (%)				
15-Asp	82.6	6.3	82.9	1.7	78.5	3.0	1.25	2.5	2~1000	0.99917
15-ADON	87.8	4.4	79.5	3.8	73.8	5.3	6.25	12.5	10~5000	0.99981
3-ADON	87.7	4.7	83.5	3.2	79.9	2.5	6.25	12.5	10~5000	0.99934
7-D-G	79.0	6.0	83.8	3.4	80.6	2.5	0.25	0.5	0.4~200	0.99981
AFB_1_	81.6	5.4	84.2	3.2	78.5	2.6	0.25	0.5	0.4~200	0.99911
AFB_2_	78.5	5.5	83.7	2.7	78.4	3.4	0.25	0.5	0.4~200	0.99923
AFG_1_	81.7	6.6	83.6	3.0	80.6	3.0	0.25	0.5	0.4~200	0.99980
AFG_2_	83.1	6.4	83.6	2.0	83.3	3.7	0.25	0.5	0.4~200	0.99929
AFM_1_	81.9	4.0	83.4	4.5	78.9	3.7	0.25	0.5	0.4~200	0.99979
AFM_2_	87.3	8.6	82.6	5.9	75.2	4.8	1.25	2.5	2~1000	0.99975
AFP_1_	80.3	6.5	81.9	4.7	77.4	4.0	1.25	2.5	2~1000	0.99912
Agro	80.3	4.8	80.2	4.2	75.3	4.6	0.25	0.5	0.4~200	0.99985
Anis	82.3	7.0	81.3	4.0	77.6	4.6	0.25	0.5	0.4~200	0.99902
Apici	82.3	4.7	83.9	2.0	79.0	3.6	1.25	2.5	2~1000	0.99904
BEA	79.2	6.8	81.7	2.4	79.3	2.9	0.25	0.5	0.4~200	0.99926
Chae	86.3	9.8	83.7	6.6	79.9	6.1	6.25	12.5	10~5000	0.99957
Che	82.0	8.6	82.3	4.6	80.4	4.8	1.25	2.5	2~1000	0.99928
CIT	77.3	6.4	79.2	7.5	74.3	8.0	1.25	2.5	2~1000	0.99902
CVD	81.9	6.3	92.5	5.0	82.8	3.6	6.25	12.5	10~5000	0.99919
CPA	81.2	7.9	77.3	5.1	77.6	5.0	1.25	2.5	2~1000	0.99903
DAS	87.8	3.7	81.8	2.8	78.3	2.7	0.25	0.5	0.4~200	0.99904
DiLy	67.0	3.6	64.0	2.3	61.6	2.5	0.25	0.5	0.4~200	0.99966
DOM	84.8	3.6	71.6	4.8	78.2	7.4	6.25	12.5	10~5000	0.99960
DON	77.7	4.5	76.7	2.5	72.1	3.2	6.25	12.5	10~5000	0.99983
ENN A	74.1	3.9	79.7	1.4	77.0	2.7	0.25	0.5	0.4~200	0.99978
ENN A_1_	76.0	3.3	82.7	3.3	82.2	3.3	0.25	0.5	0.4~200	0.99990
ENN B	82.0	3.7	84.2	3.8	82.7	3.8	0.25	0.5	0.4~200	0.99996
ENN B_1_	80.1	4.3	84.2	4.2	83.7	2.9	0.25	0.5	0.4~200	0.99981
Equi	75.0	4.2	72.2	2.9	77.4	2.3	1.25	2.5	2~1000	0.99908
EGCN	88.9	3.9	85.1	1.5	78.9	4.2	0.25	0.5	0.4~200	0.99940
EGCNN	78.7	5.5	80.7	4.0	76.4	3.8	0.25	0.5	0.4~200	0.99916
EGST	86.1	4.3	84.1	1.8	82.9	2.5	0.25	0.5	0.4~200	0.99990
EGSTN	80.9	4.4	81.0	3.0	76.0	3.0	0.25	0.5	0.4~200	0.99936
EGPT	86.8	3.8	81.7	4.7	78.8	3.0	0.25	0.5	0.4~200	0.99919
EGPTN	85.8	3.9	81.1	2.7	75.8	3.2	0.25	0.5	0.4~200	0.99979
EGSN	87.6	13.6	88.9	11.6	78.0	4.5	0.25	0.5	0.4~200	0.99933
FB_1_	78.0	7.3	70.7	5.1	77.0	2.6	1.25	2.5	2~1000	0.99999
FB_2_	79.1	7.3	76.1	2.8	73.1	5.5	1.25	2.5	2~1000	0.99983
FB_3_	63.6	8.4	73.2	2.9	72.0	2.2	1.25	2.5	2~1000	0.99997
Fum	71.2	6.2	81.8	3.8	76.9	6.7	6.25	12.5	10~5000	0.99963
FuX	89.3	4.4	81.1	3.5	76.2	2.6	6.25	12.5	10~5000	0.99948
Glio	80.4	5.4	81.7	4.1	79.7	4.3	1.25	2.5	2~1000	0.99983
Grise	82.7	5.4	83.1	3.6	79.3	2.5	0.25	0.5	0.4~200	0.99960
HT-2	78.9	7.5	81.5	7.3	80.4	3.4	6.25	12.5	10~5000	0.99936
Lyser	68.4	2.2	69.7	3.5	66.9	2.4	0.25	0.5	0.4~200	0.99923
Melea	82.0	4.3	80.0	3.9	78.2	3.2	0.25	0.5	0.4~200	0.99926
Mono	75.6	7.1	82.1	2.8	79.5	3.2	1.25	2.5	2~1000	0.99979
MPA	79.1	10.5	87.7	4.4	84.7	2.1	0.25	0.5	0.4~200	0.99907
NEO	91.4	2.9	81.3	4.3	75.5	3.0	1.25	2.5	2~1000	0.99972
O-m-Ster	81.0	4.4	83.3	2.5	81.1	2.8	1.25	2.5	2~1000	0.99904
Ostre A	90.0	5.0	89.0	4.1	81.3	6.9	6.25	12.5	10~5000	0.99941
OTA	73.6	7.0	83.4	10.3	81.0	6.4	0.25	0.5	0.4~200	0.99926
OTB	70.4	7.0	85.8	3.1	88.5	3.6	0.25	0.5	0.4~80	0.99953
OTC	77.2	5.6	79.6	3.3	78.7	1.9	0.25	0.5	0.4~200	0.99980
Oxa	85.3	3.6	79.9	3.4	76.6	4.3	0.25	0.5	0.4~200	0.99956
Pse A	89.5	4.0	82.5	4.3	79.3	4.3	1.25	2.5	2~1000	0.99977
Puro	71.1	3.5	62.9	3.2	67.1	4.8	0.25	0.5	0.4~200	0.99963
Rq C	87.0	2.7	78.5	3.0	74.5	3.2	0.25	0.5	0.4~200	0.99931
Secal A	73.5	5.3	79.3	3.4	75.4	1.0	1.25	2.5	2~1000	0.99928
Ster	77.8	10.6	82.3	4.0	80.5	2.9	0.25	0.5	0.4~200	0.99910
T2-triol	91.2	2.7	82.1	4.0	77.9	3.2	6.25	12.5	10~5000	0.99984
T-2	82.1	3.3	83.7	2.3	82.3	4.0	1.25	2.5	2~1000	0.99971
Ten	81.7	3.3	83.0	2.9	82.5	3.1	6.25	12.5	10~5000	0.99926
Wor-man	90.9	3.4	83.2	3.7	79.7	6.7	6.25	12.5	10~5000	0.99907
α-ZAL	86.0	9.8	92.6	5.6	75.9	5.1	1.25	2.5	2~1000	0.99934
α-ZEL	106.5	11.1	85.2	4.5	84.3	5.6	1.25	2.5	2~1000	0.99921
β-ZAL	92.4	9.3	105.3	3.9	89.6	5.8	1.25	2.5	2~1000	0.99906
β-ZEL	80.1	8.3	97.4	4.4	86.6	6.7	1.25	2.5	2~1000	0.99906
PAT	88.7	3.5	75.9	7.6	73.0	9.9	6.25	12.5	10~5000	0.99995
ZAN	88.7	7.5	92.3	4.0	80.6	3.5	1.25	2.5	2~1000	0.99900
ZEN	91.7	11.5	81.5	3.6	78.4	3.5	1.25	2.5	2~1000	0.99961
AME	96.2	10.7	77.3	3.6	76.0	3.8	6.25	12.5	10~1000	0.99912
AOH	84.4	9.5	90.2	7.0	83.1	4.5	6.25	12.5	10~1000	0.99868

**Table A4:** The muti-mycotoxin contamination situation of LS.

Sample	Comment	Contamination Level (μg/kg)								
		AFB_1_	AFB_2_	AFG_1_	AFG_2_	AFM_1_	AFM_2_	CIT	CPA	MPA
LS1	original shape	3.0	21.4	ND	ND	1.0	ND	ND	ND	ND
LS2	original shape	ND	ND	ND	ND	ND	ND	ND	ND	ND
LS3	original shape	ND	ND	ND	ND	1.8	ND	ND	ND	ND
LS4	original shape	4.6	1.3	ND	ND	ND	ND	ND	ND	ND
LS5	original shape	ND	ND	ND	ND	ND	ND	ND	ND	1.3
LS6	original shape	ND	ND	ND	ND	ND	ND	ND	ND	ND
LS7	original shape	1.3	ND	ND	ND	ND	ND	ND	ND	ND
LS8	original shape	ND	ND	ND	ND	ND	ND	ND	ND	ND
LS9	original shape	18.4	1.3	ND	ND	ND	ND	ND	ND	ND
LS10	original shape	ND	ND	ND	ND	ND	ND	ND	ND	ND
LS11	original shape	ND	ND	ND	ND	ND	ND	ND	ND	ND
LS12	original shape	ND	ND	ND	ND	ND	ND	ND	ND	ND
LS13	original shape	ND	ND	ND	ND	ND	ND	ND	ND	ND
LS14	original shape	31.6	1.3	ND	ND	ND	ND	ND	ND	ND
LS15	original shape	21.5	ND	ND	ND	ND	ND	ND	ND	27.8
LS16	original shape	16.4	1.3	ND	ND	ND	ND	ND	ND	75.0
LS17	original shape	ND	ND	ND	ND	ND	ND	ND	ND	ND
LS18	original shape	ND	ND	ND	ND	ND	ND	ND	ND	ND
LS19	original shape	ND	ND	ND	ND	ND	ND	ND	ND	ND
LS20	original shape	ND	ND	ND	ND	ND	ND	ND	ND	ND
LS21	powder	ND	ND	ND	ND	ND	ND	ND	406.9	ND
LS22	powder	12.6	ND	ND	ND	ND	ND	ND	1043.7	8.6
LS23	powder	14.9	ND	ND	ND	ND	ND	ND	1563.7	ND
LS24	powder, fresh	3638.6	160.8	ND	ND	47.5	ND	ND	12199.8	ND
LS25	powder, farm-grown	4445.4	351.9	17ND	8.8	189.6	19.5	376.8	19520.6	ND
LS26	powder, farm-grown	4700.6	396.5	ND	ND	155.1	29.8	ND	23574.6	ND
LS27	powder, farm-grown	ND	ND	ND	ND	ND	ND	ND	14.6	ND
LS28	Powder, moldy	ND	ND	ND	ND	ND	ND	ND	5.0	ND
LS29	Powder, discolored	ND	ND	ND	ND	ND	ND	ND	2.6	ND
Contamination rate (%)		41.4	27.6	3.4	3.4	17.2	6.9	3.4	31.0	13.8
**Sample**	**Comment**	**Contamination Level (μg/kg)**								
		**ZEN**	**BEA**	**ENN A1**	**Glio**	**O-m-Ster**	**Pse A**	**Ster**	**Ten**	
LS1	original shape	ND	ND	ND	ND	ND	ND	ND	1.3	
LS2	original shape	ND	ND	ND	ND	ND	ND	6.5	ND	
LS3	original shape	ND	ND	ND	ND	11.7	ND	ND	ND	
LS4	original shape	1.3	ND	ND	ND	ND	ND	1.3	ND	
LS5	original shape	ND	11.1	1.3	ND	ND	ND	1.3	ND	
LS6	original shape	ND	ND	1.3	ND	3.8	ND	ND	ND	
LS7	original shape	ND	ND	ND	ND	ND	ND	ND	ND	
LS8	original shape	ND	ND	ND	ND	ND	ND	8.2	ND	
LS9	original shape	ND	14.0	1.8	ND	ND	ND	ND	9.3	
LS10	original shape	ND	ND	ND	ND	ND	ND	ND	ND	
LS11	original shape	ND	ND	ND	ND	ND	ND	ND	ND	
LS12	original shape	ND	ND	ND	ND	ND	ND	ND	ND	
LS13	original shape	ND	ND	ND	ND	ND	ND	ND	ND	
LS14	original shape	ND	10.5	ND	ND	ND	ND	ND	16.6	
LS15	original shape	ND	14.3	ND	ND	ND	ND	ND	15.1	
LS16	original shape	34.1	35.5	16.2	ND	ND	ND	ND	21.1	
LS17	original shape	ND	11.6	ND	70.7	ND	ND	ND	3.7	
LS18	original shape	1.3	10.6	30.4	12.5	ND	ND	ND	ND	
LS19	original shape	1.3	27.3	3.3	8.6	ND	ND	ND	ND	
LS20	original shape	ND	15.9	1.5	ND	ND	ND	ND	ND	
LS21	powder	ND	ND	ND	ND	ND	ND	ND	ND	
LS22	powder	ND	ND	ND	ND	ND	ND	ND	ND	
LS23	powder	ND	ND	ND	ND	ND	6.4	ND	ND	
LS24	powder, fresh	ND	ND	ND	ND	11.4	ND	7.0	ND	
LS25	powder, farm-grown	ND	ND	ND	ND	158.8	ND	5.0	ND	
LS26	powder, farm-grown	ND	ND	ND	ND	118.8	ND	ND	ND	
LS27	powder, farm-grown	ND	ND	ND	ND	ND	ND	ND	ND	
LS28	Powder, moldy	ND	ND	ND	ND	ND	ND	ND	ND	
LS29	Powder, discolored	ND	ND	ND	ND	ND	ND	ND	ND	
Contamination rate (%)		13.8	31.0	24.1	10.3	17.2	3.4	20.7	20.7	

**Table A5:** The muti-mycotoxin contamination situation of LR.

Sample	Comment	Vintage Year	Contamination Level (μg/kg)								
			ZEN	DOM	DON	BEA	ENN A	ENN A1	ENN B	ENN B1	FB_1_
LR1	slice	/	ND	ND	ND	ND	ND	ND	1.3	1.3	8.3
LR2	slice	/	ND	ND	ND	ND	ND	ND	13.8	1.3	13
LR3	slice	/	2.2	ND	ND	ND	ND	ND	ND	ND	1.3
LR4	slice	/	ND	ND	ND	ND	ND	ND	ND	ND	ND
LR5	slice	/	ND	ND	ND	ND	ND	ND	ND	ND	ND
LR6	slice	/	26.9	15.1	15.1	1.3	ND	32.3	77.2	104.8	ND
LR7	slice	/	ND	ND	ND	ND	ND	ND	ND	ND	ND
LR8	slice	/	ND	ND	ND	ND	ND	ND	ND	ND	ND
LR9	slice	/	ND	ND	ND	1.3	ND	1.3	1.3	ND	4.8
LR10	slice	/	ND	ND	ND	ND	ND	ND	ND	ND	1.3
LR11	slice	/	ND	ND	ND	ND	ND	ND	ND	ND	ND
LR12	slice	/	ND	ND	ND	2.5	ND	1.3	ND	ND	14.6
LR13	slice	/	ND	ND	ND	ND	ND	ND	ND	ND	ND
LR14	slice	/	ND	ND	ND	ND	ND	ND	ND	ND	ND
LR15	slice	/	ND	ND	ND	ND	ND	ND	ND	ND	ND
LR16	slice	/	ND	ND	ND	ND	ND	ND	ND	ND	ND
LR17	slice	/	ND	ND	ND	1.3	ND	17.7	61.2	85.4	34.9
LR18	slice	/	ND	ND	ND	ND	ND	ND	ND	ND	ND
LR19	slice	/	ND	ND	ND	ND	ND	ND	ND	ND	ND
LR20	slice	/	1.3	ND	ND	ND	ND	ND	ND	ND	1.3
LR21	slice	/	94.6	ND	ND	21.4	ND	27.2	23.2	94.8	10.2
LR22	slice	/	53.1	ND	ND	ND	ND	ND	ND	ND	1.3
LR23	slice	/	ND	ND	ND	ND	ND	ND	ND	ND	1.3
LR24	slice	/	ND	ND	ND	ND	ND	ND	ND	ND	20.7
LR25	slice	/	ND	ND	ND	ND	ND	ND	ND	ND	ND
LR26	slice	/	ND	ND	ND	ND	ND	ND	ND	ND	ND
LR27	slice	/	ND	ND	ND	ND	ND	ND	ND	ND	ND
LR28	slice	/	ND	ND	ND	ND	ND	ND	ND	ND	ND
LR29	slice	/	ND	ND	ND	ND	ND	ND	ND	ND	1.3
LR30	slice	/	ND	ND	ND	1.3	ND	ND	ND	ND	8
LR31	original shape, a *	2018	ND	ND	ND	ND	ND	ND	ND	ND	ND
LR32	original shape, b	2018	39.5	ND	ND	ND	ND	ND	ND	ND	ND
LR33	original shape, c	2018	ND	ND	ND	ND	ND	ND	ND	ND	ND
LR34	original shape, d	2018	ND	ND	ND	ND	ND	ND	ND	ND	ND
LR35	original shape, d	2018	42	ND	ND	ND	ND	ND	ND	ND	ND
LR36	original shape, d	2018	ND	ND	ND	ND	ND	ND	ND	ND	ND
LR37	original shape, d	2018	ND	ND	ND	ND	ND	ND	ND	ND	ND
LR38	original shape	2019	ND	ND	ND	ND	ND	ND	ND	ND	ND
LR39	original shape, d	2019	ND	ND	ND	ND	ND	ND	ND	ND	ND
LR40	original shape, d	2019	ND	ND	ND	ND	ND	ND	ND	ND	ND
LR41	original shape, d	2019	ND	ND	ND	ND	ND	ND	ND	ND	ND
LR42	original shape, d	2019	ND	ND	ND	ND	ND	ND	ND	ND	ND
LR43	original shape, d	2019	ND	ND	ND	ND	ND	ND	ND	ND	ND
LR44	original shape, d	2019	ND	ND	ND	ND	ND	ND	ND	ND	ND
LR45	original shape, d	2019	ND	ND	ND	ND	ND	ND	ND	ND	ND
LR46	slice, d	2019	ND	ND	ND	ND	ND	ND	ND	ND	ND
LR47	slice, d	2019	ND	ND	ND	ND	ND	ND	ND	ND	ND
LR48	slice, d	2019	ND	ND	ND	ND	ND	ND	ND	ND	ND
LR49	slice, d	2019	40.4	ND	ND	ND	ND	ND	ND	ND	ND
LR50	original shape, d	2016	ND	ND	ND	ND	ND	ND	ND	ND	ND
LR51	original shape, d	2016	ND	ND	ND	ND	ND	ND	ND	ND	ND
LR52	original shape, d	2018	ND	ND	ND	ND	ND	ND	1.6	ND	ND
LR53	original shape, d	2018	ND	ND	ND	ND	ND	ND	ND	ND	ND
LR54	original shape, d	2015	ND	ND	ND	ND	ND	ND	1.1	ND	ND
LR55	original shape, d	2015	ND	ND	ND	1.1	ND	ND	ND	ND	ND
LR56	original shape, d	2015	ND	ND	ND	2	ND	ND	ND	ND	ND
LR57	original shape, d	2015	ND	ND	ND	1	ND	ND	ND	ND	ND
LR58	original shape, d	2017	ND	ND	ND	ND	ND	ND	1.7	ND	ND
LR59	original shape, d	2017	ND	ND	ND	ND	ND	ND	ND	ND	ND
LR60	original shape, c	2019	34.1	ND	ND	1.8	ND	ND	1.3	ND	ND
LR61	original shape, c	2019	ND	ND	ND	5.9	ND	ND	1.4	ND	ND
LR62	original shape, c	2015	5.3	ND	ND	90.2	15.9	38.7	119.7	90.6	ND
LR63	original shape, e	2018	ND	ND	ND	7.2	5.2	14	53.2	31.0	ND
LR64	original shape, e	2019	ND	ND	ND	483.2	2.3	18.2	166.8	84.5	ND
LR65	original shape, e	2020	ND	ND	ND	ND	5.9	31.2	188.2	100.8	ND
LR66	original shape, a	2018	ND	ND	ND	21.8	ND	ND	3.2	1.9	ND
LR67	original shape, a	2019	ND	ND	ND	ND	ND	ND	3.5	1.9	ND
LR68	original shape, a	2020	5.8	ND	ND	ND	ND	ND	1.1	ND	ND
LR69	original shape, c	2019	ND	ND	ND	3.2	ND	ND	ND	ND	ND
LR70	original shape, c	2020	ND	ND	ND	3.2	ND	ND	3.1	1.8	ND
LR71	original shape, c	2019	ND	ND	ND	ND	ND	ND	1.4	ND	ND
LR72	original shape, c	2019	ND	ND	ND	ND	ND	ND	ND	ND	ND
LR73	original shape, c	2020	ND	ND	ND	ND	ND	ND	1.7	1.1	ND
LR74	original shape, b	2017	17.4	ND	ND	4.9	ND	ND	1	ND	ND
LR75	original shape, b	2020	ND	ND	ND	10.8	ND	ND	2.1	1.5	ND
LR76	original shape, b	2018	ND	ND	ND	ND	ND	ND	ND	ND	ND
LR77	original shape, b	2019	ND	ND	ND	2.1	ND	ND	1.9	ND	ND
Contamination rate (%)			15.6	1.3	1.3	26.0	5.2	11.7	31.2	18.2	18.2
**Sample**	**Comment**	**Vintage year**	**Contamination level (μg/kg)**								
			**FB_2_**	**Melea**	**OTA**	**MPA**	**Pse A**	**CIT**	**AOH**	**AME**	**DiLy**
LR1	slice	/	ND	ND	3.2	ND	15.3	4.6	ND	ND	ND
LR2	slice	/	ND	ND	ND	ND	ND	1.3	ND	ND	ND
LR3	slice	/	ND	ND	ND	1.3	ND	ND	ND	1.3	ND
LR4	slice	/	ND	ND	ND	ND	ND	ND	ND	ND	ND
LR5	slice	/	ND	ND	ND	ND	ND	ND	ND	ND	ND
LR6	slice	/	ND	ND	ND	1.3	ND	ND	ND	ND	ND
LR7	slice	/	ND	ND	ND	ND	ND	ND	ND	ND	ND
LR8	slice	/	ND	ND	ND	ND	ND	ND	ND	14.5	ND
LR9	slice	/	14.4	ND	ND	ND	ND	ND	ND	ND	ND
LR10	slice	/	ND	ND	2.4	ND	4.6	ND	ND	ND	ND
LR11	slice	/	ND	ND	ND	ND	ND	ND	ND	ND	ND
LR12	slice	/	6.3	ND	ND	ND	ND	ND	ND	ND	ND
LR13	slice	/	ND	ND	ND	ND	ND	ND	ND	ND	ND
LR14	slice	/	ND	ND	ND	ND	ND	ND	ND	19.2	ND
LR15	slice	/	ND	ND	ND	ND	ND	ND	ND	ND	ND
LR16	slice	/	ND	ND	ND	107.0	ND	ND	ND	ND	ND
LR17	slice	/	10.2	ND	ND	99.8	ND	ND	ND	ND	ND
LR18	slice	/	ND	ND	ND	ND	ND	ND	ND	ND	ND
LR19	slice	/	ND	ND	ND	ND	ND	ND	ND	ND	ND
LR20	slice	/	12.4	ND	ND	ND	ND	ND	ND	1.3	ND
LR21	slice	/	ND	ND	ND	1.3	ND	ND	ND	ND	ND
LR22	slice	/	ND	ND	6.4	ND	ND	ND	ND	ND	ND
LR23	slice	/	1.3	ND	ND	ND	ND	ND	ND	ND	ND
LR24	slice	/	8.9	5.7	ND	1.3	ND	ND	ND	ND	ND
LR25	slice	/	ND	ND	ND	ND	ND	ND	ND	ND	ND
LR26	slice	/	ND	ND	ND	ND	ND	ND	ND	ND	ND
LR27	slice	/	ND	ND	ND	ND	ND	ND	ND	ND	ND
LR28	slice	/	1.3	ND	ND	ND	ND	ND	ND	ND	ND
LR29	slice	/	ND	24.8	ND	ND	ND	ND	ND	15.9	ND
LR30	slice	/	1.3	1.3	ND	ND	ND	ND	ND	ND	ND
LR31	original shape, a *	2018	ND	ND	ND	ND	ND	ND	ND	ND	ND
LR32	original shape, b	2018	ND	ND	ND	ND	ND	ND	ND	ND	ND
LR33	original shape, c	2018	ND	ND	ND	ND	ND	ND	ND	ND	ND
LR34	original shape, d	2018	ND	ND	ND	ND	ND	ND	ND	ND	ND
LR35	original shape, d	2018	ND	ND	ND	ND	ND	ND	ND	ND	ND
LR36	original shape, d	2018	ND	ND	ND	ND	ND	ND	ND	ND	ND
LR37	original shape, d	2018	ND	ND	ND	ND	ND	ND	ND	ND	ND
LR38	original shape	2019	ND	ND	ND	ND	ND	ND	ND	ND	ND
LR39	original shape, d	2019	ND	ND	ND	ND	ND	ND	ND	ND	ND
LR40	original shape, d	2019	ND	ND	ND	ND	ND	ND	ND	ND	ND
LR41	original shape, d	2019	ND	ND	ND	ND	ND	ND	ND	ND	ND
LR42	original shape, d	2019	ND	ND	ND	ND	ND	ND	ND	ND	ND
LR43	original shape, d	2019	ND	ND	ND	ND	ND	ND	ND	ND	ND
LR44	original shape, d	2019	ND	ND	ND	ND	ND	ND	ND	ND	ND
LR45	original shape, d	2019	ND	ND	ND	ND	ND	ND	ND	ND	ND
LR46	slice, d	2019	ND	ND	ND	ND	ND	ND	ND	ND	ND
LR47	slice, d	2019	ND	ND	ND	ND	ND	ND	ND	ND	ND
LR48	slice, d	2019	ND	ND	ND	ND	ND	ND	ND	ND	ND
LR49	slice, d	2019	ND	ND	ND	ND	ND	ND	ND	ND	ND
LR50	original shape, d	2016	ND	ND	ND	ND	ND	ND	ND	ND	ND
LR51	original shape, d	2016	ND	ND	ND	ND	ND	ND	ND	ND	ND
LR52	original shape, d	2018	ND	ND	ND	ND	ND	ND	ND	ND	ND
LR53	original shape, d	2018	ND	ND	ND	ND	ND	ND	ND	ND	ND
LR54	original shape, d	2015	ND	ND	ND	ND	ND	ND	ND	ND	2.5
LR55	original shape, d	2015	ND	ND	ND	ND	ND	ND	ND	ND	ND
LR56	original shape, d	2015	ND	ND	ND	ND	ND	ND	ND	ND	ND
LR57	original shape, d	2015	ND	ND	ND	ND	ND	ND	ND	ND	ND
LR58	original shape, d	2017	ND	ND	ND	ND	ND	ND	ND	ND	ND
LR59	original shape, d	2017	ND	ND	ND	ND	ND	ND	ND	ND	ND
LR60	original shape, c	2019	ND	ND	ND	ND	ND	ND	ND	ND	ND
LR61	original shape, c	2019	ND	ND	ND	ND	ND	ND	ND	ND	ND
LR62	original shape, c	2015	ND	ND	ND	ND	ND	ND	ND	ND	ND
LR63	original shape, e	2018	ND	5.1	ND	ND	ND	ND	ND	ND	ND
LR64	original shape, e	2019	ND	ND	ND	ND	ND	ND	85.5	ND	ND
LR65	original shape, e	2020	ND	ND	ND	ND	ND	ND	ND	ND	ND
LR66	original shape, a	2018	ND	ND	ND	ND	ND	ND	ND	ND	ND
LR67	original shape, a	2019	ND	ND	ND	ND	ND	ND	ND	ND	ND
LR68	original shape, a	2020	ND	ND	ND	ND	ND	ND	ND	ND	ND
LR69	original shape, c	2019	ND	ND	ND	ND	ND	ND	ND	ND	ND
LR70	original shape, c	2020	ND	ND	ND	ND	ND	ND	ND	ND	ND
LR71	original shape, c	2019	ND	ND	ND	ND	ND	ND	ND	ND	ND
LR72	original shape, c	2019	ND	ND	ND	ND	ND	ND	ND	ND	ND
LR73	original shape, c	2020	ND	ND	ND	ND	ND	ND	ND	ND	ND
LR74	original shape, b	2017	ND	ND	ND	ND	ND	ND	ND	ND	ND
LR75	original shape, b	2020	ND	ND	ND	ND	ND	ND	ND	ND	ND
LR76	original shape, b	2018	ND	1.1	ND	ND	ND	ND	ND	ND	ND
LR77	original shape, b	2019	ND	ND	ND	ND	ND	ND	ND	ND	ND
Contamination rate (%)			10.4	6.5	3.9	7.8	2.6	2.6	1.3	6.5	1.3

*: a: Jilin, b: Ningxia, c: Neimenggu, d: Xinjiang, e: Gansu.

**Table A6:** The muti-mycotoxin contamination situation of CP.

Sample	Comment	Contamination Level (μg/kg)				
		BEA	ENN A1	ENN B	ENN B1	MPA
CP1	original shape	ND	ND	ND	ND	6.7
CP2	original shape	ND	ND	ND	ND	ND
CP3	original shape	ND	ND	ND	ND	9.8
CP4	original shape	ND	ND	ND	ND	14
CP5	original shape	ND	ND	ND	ND	ND
CP6	original shape	ND	ND	ND	ND	ND
CP7	original shape	ND	ND	ND	ND	25.7
CP8	original shape	ND	ND	ND	ND	ND
CP9	original shape	ND	ND	ND	ND	24.1
CP10	original shape	ND	ND	ND	ND	ND
CP11	original shape	ND	ND	ND	ND	ND
CP12	original shape	ND	ND	ND	ND	ND
CP13	original shape	ND	ND	ND	ND	15.2
CP14	original shape	ND	ND	ND	ND	16
CP15	original shape	6.9	3	19.3	6.1	ND
CP16	original shape	ND	ND	ND	ND	17.9
CP17	original shape	ND	ND	ND	ND	ND
CP18	original shape	ND	ND	ND	ND	14.2
CP19	original shape	ND	ND	ND	ND	ND
CP20	original shape	14	2.7	15.8	4.7	11.4
CP21	original shape	ND	ND	ND	ND	ND
CP22	original shape	ND	ND	ND	ND	15.8
CP23	original shape	ND	ND	ND	ND	ND
CP24	original shape	ND	ND	ND	ND	ND
CP25	original shape	ND	ND	ND	ND	ND
CP26	original shape	ND	ND	ND	ND	20.5
CP27	original shape	ND	ND	ND	ND	18.3
CP28	original shape	ND	ND	ND	ND	ND
CP29	original shape	ND	ND	ND	ND	16
CP30	original shape	ND	ND	ND	ND	ND
CP31	original shape	ND	ND	ND	ND	22.3
CP32	original shape	ND	ND	ND	ND	ND
CP33	original shape	ND	ND	ND	ND	12
CP34	original shape	8.7	2.7	30.5	13.2	22.8
CP35	original shape	ND	ND	ND	ND	ND
CP36	original shape	ND	ND	ND	ND	ND
CP37	original shape	ND	ND	ND	ND	16.8
CP38	original shape	ND	ND	ND	ND	34.4
CP39	original shape	ND	ND	ND	ND	ND
CP40	original shape	ND	ND	ND	ND	14.2
CP41	original shape	ND	ND	ND	ND	ND
CP42	original shape	ND	ND	ND	ND	ND
CP43	original shape	ND	ND	ND	ND	21
CP44	original shape	ND	ND	ND	ND	ND
CP45	original shape	ND	ND	ND	ND	15.8
CP46	original shape	ND	ND	ND	ND	ND
CP47	original shape	ND	ND	ND	ND	15
CP48	original shape	12.1	1.2	16.4	3.7	ND
CP49	original shape	10.5	1.6	22.7	5.1	22.0
CP50	original shape	ND	ND	ND	ND	ND
CP51	original shape	ND	ND	ND	ND	16.3
CP52	original shape	ND	ND	ND	ND	ND
CP53	original shape	ND	ND	ND	ND	10.5
CP54	original shape	ND	ND	ND	ND	ND
CP55	original shape	ND	ND	ND	ND	11.9
CP56	original shape	ND	ND	ND	ND	21.4
CP57	original shape	ND	ND	ND	ND	16.5
CP58	original shape	ND	ND	ND	ND	ND
CP59	original shape	ND	ND	ND	ND	ND
CP60	original shape	ND	ND	ND	ND	ND
CP61	original shape	ND	ND	ND	ND	ND
CP62	original shape	ND	ND	ND	ND	18.3
CP63	original shape	ND	ND	ND	ND	ND
CP64	original shape	5.5	7.3	65.8	20.0	16.8
CP65	original shape	ND	ND	ND	ND	ND
CP66	original shape	ND	ND	ND	ND	22.8
CP67	original shape	ND	ND	ND	ND	ND
CP68	original shape	ND	ND	ND	ND	12.1
CP69	original shape	ND	ND	ND	ND	22.4
CP70	original shape	ND	ND	ND	ND	ND
CP71	original shape	ND	ND	ND	ND	ND
CP72	original shape	ND	ND	ND	ND	16.7
CP73	original shape	ND	ND	ND	ND	ND
CP74	original shape	ND	ND	ND	ND	16.8
CP75	original shape	ND	ND	ND	ND	ND
CP76	original shape	ND	ND	ND	ND	ND
CP77	original shape	ND	ND	ND	ND	20.4
CP78	original shape	ND	ND	ND	ND	ND
CP79	original shape	ND	ND	ND	ND	15.6
CP80	original shape	ND	ND	ND	ND	ND
CP81	original shape	ND	ND	ND	ND	19.9
CP82	original shape	ND	ND	ND	ND	ND
CP83	original shape	ND	ND	ND	ND	ND
CP84	original shape	ND	ND	ND	ND	26.7
CP85	original shape	ND	ND	ND	ND	ND
CP86	original shape	ND	ND	ND	ND	ND
CP87	original shape	ND	ND	ND	ND	ND
CP88	original shape	ND	ND	ND	ND	ND
CP89	original shape	ND	ND	ND	ND	32.3
CP90	original shape	ND	ND	ND	ND	ND
CP91	original shape	ND	ND	ND	ND	ND
CP92	original shape	ND	ND	ND	ND	11.8
CP93	original shape	6.2	15.4	167.2	34.6	12.4
CP94	original shape	5.1	7.3	81.3	20.2	14.5
CP95	original shape	8.8	3.7	35.9	11	21.8
CP96	original shape	ND	ND	ND	ND	ND
CP97	original shape	ND	ND	ND	ND	ND
CP98	original shape	ND	ND	ND	ND	ND
CP99	original shape	ND	ND	ND	ND	33
CP100	original shape	11.5	11.7	100.4	25.6	21.7
CP101	original shape	ND	ND	ND	ND	ND
CP102	original shape	ND	ND	ND	ND	ND
CP103	original shape	ND	ND	ND	ND	ND
CP104	original shape	ND	ND	ND	ND	ND
CP105	original shape	ND	ND	ND	ND	ND
CP106	original shape	ND	ND	ND	ND	ND
CP107	original shape	ND	ND	ND	ND	ND
CP108	original shape	ND	ND	ND	ND	ND
CP109	original shape	ND	ND	ND	ND	ND
CP110	original shape	ND	ND	ND	ND	ND
CP111	original shape	ND	ND	ND	ND	ND
CP112	original shape	10.3	ND	10.7	3.3	ND
CP113	powder	ND	ND	ND	ND	ND
CP114	powder	ND	ND	ND	ND	ND
CP115	powder	ND	ND	ND	ND	ND
CP116	original shape	ND	ND	ND	ND	ND
CP117	original shape	ND	ND	ND	ND	ND
CP118	original shape	ND	ND	ND	ND	ND
CP119	original shape	ND	ND	ND	ND	ND
CP120	original shape	ND	ND	ND	ND	ND
CP121	original shape	ND	ND	ND	ND	ND
CP122	original shape	ND	ND	ND	ND	ND
CP123	original shape	ND	ND	ND	ND	ND
CP124	original shape	ND	ND	ND	ND	ND
CP125	original shape	ND	ND	ND	ND	ND
CP126	original shape	ND	ND	ND	ND	ND
CP127	original shape	ND	ND	ND	ND	ND
CP128	original shape	ND	ND	ND	ND	ND
CP129	original shape	ND	ND	ND	ND	ND
CP130	original shape	ND	ND	ND	ND	ND
CP131	original shape	ND	ND	ND	ND	ND
Contamination rate (%)		8.4	7.6	8.4	8.4	35.9

**Table A7:** The muti-mycotoxin contamination situation of CS.

Sample	Comment	Contamination Level (μg/kg)								
		15-Asp	3-ADON	Apici	BEA	DON	DAS	Equi	FB_1_	FB_2_
CS1	original shape	21.3	ND	ND	ND	ND	37.1	ND	24.3	10.5
CS2	original shape	2.2	ND	ND	ND	ND	2.3	ND	13.2	3.0
CS3	original shape	6.8	ND	ND	0.2	100.4	7.3	ND	353.0	50.5
CS4	original shape	1.0	ND	ND	ND	ND	1.9	ND	91.5	9.0
CS5	original shape	1.0	ND	ND	ND	41.7	2.6	ND	98.0	5.7
CS6	original shape	1.6	20.6	ND	ND	32.3	2.8	ND	ND	ND
CS7	original shape	1.0	ND	ND	2.0	1.0	2.7	1.0	ND	ND
CS8	original shape	1.0	ND	ND	ND	1.0	1.5	ND	5.0	ND
CS9	original shape	1.0	ND	ND	ND	1.0	0.7	ND	96.3	5.5
CS10	original shape	5.2	ND	0.6	ND	12.3	3.8	ND	36.4	ND
CS11	original shape	4.6	ND	ND	ND	422.3	3.0	ND	ND	ND
CS12	original shape	2.1	ND	ND	ND	ND	9.4	ND	ND	ND
CS13	original shape	ND	ND	ND	ND	21.5	1.2	ND	18.3	2.3
CS14	original shape	8.8	ND	ND	ND	ND	4.4	ND	4.1	ND
CS15	original shape	10.5	ND	ND	ND	ND	9.6	ND	56.4	10.7
CS16	original shape	ND	ND	ND	ND	ND	ND	ND	5.6	ND
CS17	original shape	ND	ND	ND	3.1	ND	ND	ND	ND	7.1
CS18	original shape	ND	ND	ND	11.1	ND	ND	ND	18.1	19.6
CS19	original shape	ND	ND	ND	ND	ND	ND	ND	ND	3.9
CS20	original shape	ND	ND	ND	ND	ND	ND	ND	11.1	8.4
CS21	original shape	ND	ND	ND	5.1	ND	ND	ND	3.2	1.1
CS22	original shape	ND	ND	ND	46.2	ND	ND	ND	2.9	ND
CS23	original shape	ND	ND	ND	4.5	ND	ND	ND	23.5	2.4
CS24	original shape	ND	ND	ND	18.6	ND	ND	ND	10.1	1.1
CS25	original shape	ND	ND	ND	13.7	ND	ND	ND	54.6	23.4
CS26	original shape	ND	ND	ND	13.9	ND	ND	ND	26.6	23.0
CS27	original shape	ND	ND	ND	ND	ND	ND	ND	3.9	ND
CS28	original shape	ND	ND	ND	14.2	ND	ND	ND	172.8	38.1
CS29	original shape	ND	ND	ND	50.0	ND	ND	ND	20.4	3.0
CS30	original shape	ND	ND	ND	5.8	ND	ND	ND	5.2	7.4
CS31	original shape	ND	ND	ND	ND	ND	ND	ND	ND	ND
CS32	original shape	ND	ND	ND	ND	ND	ND	ND	ND	ND
CS33	original shape	ND	ND	ND	ND	ND	ND	ND	ND	ND
CS34	original shape	ND	ND	ND	11.7	ND	ND	ND	21.9	10.3
CS35	original shape	ND	ND	ND	ND	ND	ND	ND	ND	ND
CS36	original shape	ND	ND	ND	2.1	ND	ND	ND	73.4	13.9
CS37	original shape	ND	ND	ND	ND	ND	ND	ND	ND	ND
CS38	original shape	ND	ND	ND	ND	ND	ND	ND	ND	ND
CS39	original shape	ND	ND	ND	ND	ND	ND	ND	4.1	ND
CS40	original shape	ND	ND	ND	ND	ND	ND	ND	16.2	5.5
CS41	original shape	ND	ND	ND	4.4	ND	ND	ND	43.0	12.6
CS42	original shape	ND	ND	ND	109.9	ND	ND	ND	69.8	17.5
CS43	original shape	ND	ND	ND	56.2	ND	ND	ND	55.7	53.9
CS44	original shape	ND	ND	ND	ND	ND	ND	ND	ND	ND
CS45	original shape	ND	ND	ND	ND	ND	ND	ND	12.4	2.2
CS46	original shape	ND	ND	ND	111.1	ND	ND	ND	54.4	13.2
CS47	original shape	ND	ND	ND	ND	ND	ND	ND	ND	ND
Contamination rate (%)		29.8	2.1	2.1	40.4	19.1	31.9	2.1	70.2	59.6
**Sample**	**comment**	**Contamination level (μg/kg)**								
		**FB_3_**	**FuX**	**MPA**	**ZEN**	**ZAN**	**α-ZEL**	**β-ZAL**	**β-ZEL**	**CVD**
CS1	original shape	2.4	ND	ND	ND	ND	ND	ND	ND	ND
CS2	original shape	6.4	ND	ND	ND	ND	ND	ND	ND	ND
CS3	original shape	27.2	50.8	ND	59.1	23.0	21.7	22.5	22.5	ND
CS4	original shape	ND	ND	ND	53.4	37.2	35.9	35.8	35.6	ND
CS5	original shape	8.3	ND	24.5	62.0	27.1	51.2	71.8	27.0	ND
CS6	original shape	ND	1.0	ND	10.0	ND	ND	ND	ND	ND
CS7	original shape	ND	ND	ND	ND	ND	ND	ND	ND	ND
CS8	original shape	ND	ND	ND	8.5	ND	ND	ND	ND	ND
CS9	original shape	ND	36.7	ND	9.6	ND	ND	ND	ND	ND
CS10	original shape	ND	21.0	ND	11.2	ND	ND	ND	ND	ND
CS11	original shape	ND	52.2	ND	9.4	ND	ND	ND	ND	ND
CS12	original shape	ND	29.3	ND	96.7	ND	ND	ND	ND	ND
CS13	original shape	ND	13.6	ND	9.8	ND	ND	ND	ND	ND
CS14	original shape	ND	ND	ND	10.8	ND	ND	ND	ND	ND
CS15	original shape	10.0	ND	ND	29.1	ND	ND	ND	ND	25.7
CS16	original shape	ND	ND	ND	12.9	ND	ND	ND	ND	ND
CS17	original shape	ND	ND	ND	ND	ND	ND	ND	ND	ND
CS18	original shape	ND	ND	ND	ND	ND	ND	ND	ND	ND
CS19	original shape	ND	ND	ND	69.1	3.8	ND	ND	ND	ND
CS20	original shape	ND	ND	ND	96.8	2.3	ND	ND	ND	ND
CS21	original shape	ND	ND	ND	ND	ND	ND	ND	ND	ND
CS22	original shape	ND	ND	ND	ND	ND	ND	ND	ND	ND
CS23	original shape	ND	ND	ND	5.6	ND	ND	ND	ND	ND
CS24	original shape	ND	ND	ND	ND	ND	ND	ND	ND	ND
CS25	original shape	ND	ND	ND	ND	ND	ND	ND	ND	ND
CS26	original shape	ND	ND	ND	ND	ND	ND	ND	ND	ND
CS27	original shape	ND	ND	ND	14.5	ND	ND	ND	ND	ND
CS28	original shape	ND	ND	ND	77.5	2.0	ND	ND	ND	ND
CS29	original shape	ND	ND	ND	ND	ND	ND	ND	ND	ND
CS30	original shape	ND	ND	ND	6.6	ND	ND	ND	ND	ND
CS31	original shape	ND	ND	ND	105.1	1.2	ND	ND	ND	ND
CS32	original shape	ND	ND	ND	28.8	1.0	ND	ND	ND	ND
CS33	original shape	ND	ND	ND	ND	ND	ND	ND	ND	ND
CS34	original shape	ND	ND	ND	81.4	3.8	ND	ND	ND	ND
CS35	original shape	ND	ND	ND	65.4	2.1	ND	ND	ND	ND
CS36	original shape	ND	ND	ND	ND	ND	ND	ND	ND	ND
CS37	original shape	ND	ND	ND	ND	ND	ND	ND	ND	ND
CS38	original shape	ND	ND	ND	ND	ND	ND	ND	ND	ND
CS39	original shape	ND	ND	ND	13.7	ND	ND	ND	ND	ND
CS40	original shape	ND	ND	ND	49.5	ND	ND	ND	ND	ND
CS41	original shape	ND	ND	ND	206.9	6.0	ND	ND	ND	ND
CS42	original shape	ND	ND	ND	4.1	ND	ND	ND	ND	ND
CS43	original shape	ND	ND	ND	ND	ND	ND	ND	ND	ND
CS44	original shape	ND	ND	ND	ND	ND	ND	ND	ND	ND
CS45	original shape	ND	ND	ND	13.2	ND	ND	ND	ND	ND
CS46	original shape	ND	ND	ND	ND	ND	ND	ND	ND	ND
CS47	original shape	ND	ND	ND	ND	ND	ND	ND	ND	ND
Contamination rate (%)		10.6	14.9	2.1	59.6	23.4	6.4	6.4	6.4	2.1
**Sample**	**comment**	**Contamination level (μg/kg)**								
		**Grise**	**CPA**	**7-D-G**	**AFB_1_**	**AFB_2_**	**OTA**	**Ster**	**Glio**	**Ten**
CS1	original shape	ND	13.3	ND	0.2	ND	ND	ND	ND	1.7
CS2	original shape	ND	3.1	ND	ND	ND	ND	ND	ND	ND
CS3	original shape	ND	ND	ND	0.6	ND	3.2	ND	ND	0.5
CS4	original shape	ND	ND	ND	0.4	ND	ND	ND	ND	0.2
CS5	original shape	ND	ND	ND	5.1	2.0	ND	0.8	ND	0.2
CS6	original shape	ND	ND	ND	ND	ND	ND	ND	ND	ND
CS7	original shape	ND	ND	ND	ND	ND	ND	ND	ND	ND
CS8	original shape	ND	ND	ND	ND	ND	ND	ND	ND	ND
CS9	original shape	ND	ND	ND	0.4	ND	ND	ND	1.0	ND
CS10	original shape	ND	ND	54.4	ND	ND	ND	ND	57.8	ND
CS11	original shape	1.8	ND	ND	4.9	0.3	ND	ND	ND	ND
CS12	original shape	ND	ND	ND	ND	ND	ND	ND	ND	ND
CS13	original shape	ND	ND	ND	ND	ND	ND	ND	ND	ND
CS14	original shape	ND	ND	ND	0.1	ND	ND	ND	ND	ND
CS15	original shape	ND	ND	ND	ND	ND	ND	ND	ND	ND
CS16	original shape	ND	ND	ND	0.3	ND	ND	ND	ND	ND
CS17	original shape	ND	ND	ND	ND	ND	ND	ND	ND	ND
CS18	original shape	ND	ND	ND	ND	ND	ND	ND	ND	ND
CS19	original shape	ND	ND	ND	ND	ND	ND	ND	ND	ND
CS20	original shape	ND	ND	ND	1.0	ND	ND	ND	ND	ND
CS21	original shape	ND	ND	ND	ND	ND	ND	ND	ND	ND
CS22	original shape	ND	ND	ND	ND	ND	ND	ND	ND	ND
CS23	original shape	ND	ND	ND	ND	ND	ND	ND	ND	ND
CS24	original shape	ND	ND	ND	ND	ND	ND	ND	ND	ND
CS25	original shape	ND	ND	ND	ND	ND	ND	ND	ND	ND
CS26	original shape	ND	ND	ND	ND	ND	ND	ND	ND	ND
CS27	original shape	ND	ND	ND	ND	ND	ND	ND	ND	ND
CS28	original shape	ND	ND	ND	ND	ND	ND	ND	ND	ND
CS29	original shape	ND	ND	ND	ND	ND	ND	ND	ND	ND
CS30	original shape	ND	ND	ND	ND	ND	ND	ND	ND	ND
CS31	original shape	ND	ND	ND	ND	ND	ND	ND	ND	ND
CS32	original shape	ND	ND	ND	ND	ND	ND	ND	ND	ND
CS33	original shape	ND	ND	ND	ND	ND	ND	ND	ND	ND
CS34	original shape	ND	ND	ND	ND	ND	ND	ND	ND	ND
CS35	original shape	ND	ND	ND	ND	ND	ND	ND	ND	ND
CS36	original shape	ND	ND	ND	ND	ND	ND	ND	ND	ND
CS37	original shape	ND	ND	ND	ND	ND	ND	ND	ND	ND
CS38	original shape	ND	ND	ND	ND	ND	ND	ND	ND	ND
CS39	original shape	ND	ND	ND	ND	ND	ND	ND	ND	ND
CS40	original shape	ND	ND	ND	3.3	1.2	ND	ND	ND	ND
CS41	original shape	ND	ND	ND	ND	ND	ND	ND	ND	ND
CS42	original shape	ND	ND	ND	2.4	ND	ND	ND	ND	ND
CS43	original shape	ND	ND	ND	1.1	ND	ND	ND	ND	ND
CS44	original shape	ND	ND	ND	ND	ND	ND	ND	ND	ND
CS45	original shape	ND	ND	ND	ND	0.7	ND	ND	ND	ND
CS46	original shape	ND	ND	ND	ND	ND	ND	ND	ND	ND
CS47	original shape	ND	ND	ND	ND	ND	ND	ND	ND	ND
Contamination rate (%)		2.1	4.3	2.1	25.5	8.5	2.1	2.1	4.3	8.5

**Table A8:** Source and purity of 73 reference standards.

No.	Mycotoxin	Lot.	Purity	Company
1	15-Acetoxyscirpenol	AS466216	99%	Apollo Scientific, Stockport, UK
2	15-Acetyldeoxynivalenol	L14261A	99.9%	Romer Labs, Tullin, Austria
3	3-Acetyldeoxynivalenol	SZBD036XV	98.3%	Sigma-Aldrich, Laramie, USA
4	7-Dechloro Griseofulvin	10-KPA-158-4	98.85%	Toronto Research Chemicals, Toronto, Canada
5	Aflatoxin B_1_	LKB0P75	98%	J&K Scientific, Shanghai, China
6	Aflatoxin B_2_	L260Q63	98%	J&K Scientific, Shanghai, China
7	Aflatoxin G_1_	282095	98%	J&K Scientific, Shanghai, China
8	Aflatoxin G_2_	LQ10Q76	98.72%	J&K Scientific, Shanghai, China
9	Aflatoxin M_1_	2-AJK-95-1	95%	Toronto Research Chemicals, Toronto, Canada
10	Aflatoxin M_2_	LB40Q56	95%	J&K Scientific, Shanghai, China
11	Aflatoxin P_1_	2-BSR-92-3	98%	Toronto Research Chemicals, Toronto, Canada
12	Agroclavine	L13212A	98.2%	Chiron AS, Trondheim, Norway
13	Anisomycin	LDC0Q85	99.04%	J&K Scientific, Shanghai, China
14	Apicidin	3-PQY-12-1	96%	Toronto Research Chemicals, Toronto, Canada
15	Beauvericin	LHB0P07	99%	J&K Scientific, Shanghai, China
16	Chaetocin	124M4012V	98%	Sigma-Aldrich, Laramie, USA
17	Chetomin	367820	97.17%	International Laboratory USA, San Bruno, USA
18	Citrinin	LH40O33	98.5	J&K Scientific, Shanghai, China
19	Citreoviridin	L280Q47	97.17%	J&K Scientific, Shanghai, China
20	Cyclopiazonic acid	085M4081V	98%	Sigma-Aldrich, Laramie, USA
21	Diacetoxyscirpenol	L280Q35	99%	J&K Scientific, Shanghai, China
22	Dihydrolysergamide	3-NAV-62-1	98%	Toronto Research Chemicals, Toronto, Canada
23	Deepoxy-deoxynivalenol	L16103A	98%	Chiron AS, Trondheim, Norway
24	Deoxynivalenol	LR10Q103	99.83%	J&K Scientific, Shanghai, China
25	Enniatin A	AL26.129	99%	BioAustralis, New South Wales, Australia
26	Enniatin A1	AL26.21	99%	BioAustralis, New South Wales, Australia
27	Enniatin B	AL26.135	99%	BioAustralis, New South Wales, Australia
28	Enniatin B1	AL23.115	99%	BioAustralis, New South Wales, Australia
29	Equisetin	2-LWJ-95-1	93.06%	Toronto Research Chemicals, Toronto, Canada
30	Ergocornine	L14331F	97.8%	Chiron AS, Trondheim, Norway
31	Ergocorninine	L15212A	97.8%	Chiron AS, Trondheim, Norway
32	Ergocristine	LK70P83	99%	Chiron AS, Trondheim, Norway
33	Ergocristinine	L15071E	99%	Chiron AS, Trondheim, Norway
34	Ergocryptine	L14331E	97.6%	Chiron AS, Trondheim, Norway
35	Ergocryptinine	L15071D	97.6%	Chiron AS, Trondheim, Norway
36	Ergosine	L15282E	99.9%	Chiron AS, Trondheim, Norway
37	Fumonisin B_1_	SZBF083XV	98.5%	Sigma-Aldrich, Laramie, USA
38	Fumonisin B_2_	SZBF089XV	98.5%	Sigma-Aldrich, Laramie, USA
39	Fumonisin B_3_	SZBE295XV	98.5%	Sigma-Aldrich, Laramie, USA
40	Fumagillin	LK70P67	98%	J&K Scientific, Shanghai, China
41	Fusarenon X	L14141F	99.9%	Romer Labs, Tullin, Austria
42	Gliotoxin	LJ50Q77	99%	J&K Scientific, Shanghai, China
43	Griseofulvin	L5C0O27	99.6%	J&K Scientific, Shanghai, China
44	HT-2 toxin	SZBA287X	99.9%	Sigma-Aldrich, Laramie, USA
45	Lysergamide	1-GAC-130-2	99.6%	Toronto Research Chemicals, Toronto, Canada
46	Meleagrin	2-LXM-37-1	97%	Toronto Research Chemicals, Toronto, Canada
47	Monocerin	AS467363	99.9%	Apollo Scientific, Stockport, UK
48	Mycophenolic Acid	LJ20O39	98%	J&K Scientific, Shanghai, China
49	Neosolaniol	SZBD144XV	99.3%	Sigma-Aldrich, Laramie, USA
50	O-methylsterigmatocystin	00013645-201	99%	ChromaDex Standards, Irvine, USA
51	Ostreogrycin A	361047	99%	International Laboratory USA, San Bruno, USA
52	Ochratoxin A	5-JKS-45-3	98%	Toronto Research Chemicals, Toronto, Canada
53	Ochratoxin B	LAC0P30	99%	J&K Scientific, Shanghai, China
54	Ochratoxin C	5-UKS-34-1	98%	Toronto Research Chemicals, Toronto, Canada
55	Oxaline	AS463714	92.87%	Apollo Scientific, Stockport, UK
56	Pseurotin A	3-PQY-14-1	95%	Toronto Research Chemicals, Toronto, Canada
57	Puromycin	00016435-939	99%	ChromaDex Standards, Irvine, USA
58	Roquefortine C	AS467985	98%	Apollo Scientific, Stockport, UK
59	Secalonic acid D	L15471S	99%	Chiron AS, Trondheim, Norway
60	Sterigmatocystin	SZBF020XV	99.3%	Sigma-Aldrich, Laramie, USA
61	T-2-triol	L15354A	98.2%	Chiron AS, Trondheim, Norway
62	T-2 toxin	2J0A02	99%	Pribolab, Qingdao, China
63	Tentoxin	1I1J28	99%	Pribolab, Qingdao, China
64	Wortmannin	L6B0P68	98%	J&K Scientific, Shanghai, China
65	α-zearalanol	086K4024	97%	Sigma-Aldrich, Laramie, USA
66	α-zearalenol	056W4021V	97%	Sigma-Aldrich, Laramie, USA
67	β-zearalanol	115K4037	98%	Sigma-Aldrich, Laramie, USA
68	β-zearalenol	115M4019V	98%	Sigma-Aldrich, Laramie, USA
69	Patulin	LAS467986	99%	Apollo Scientific, Stockport, UK
70	Zearalanone	4-RNP-61-1	96%	Toronto Research Chemicals, Toronto, Canada
71	Zearalenone	SZBC355XV	99.3%	Sigma-Aldrich, Laramie, USA
72	Alternariol-methylether	045M4017V	96%	Sigma-Aldrich, Laramie, USA
73	Alternariol	084M4167V	96%	Sigma-Aldrich, Laramie, USA
